# Whole-exome and whole-genome sequencing of 1064 individuals with type 1 diabetes reveals novel genes for diabetic kidney disease

**DOI:** 10.1007/s00125-024-06241-1

**Published:** 2024-08-06

**Authors:** Jani K. Haukka, Anni A. Antikainen, Erkka Valo, Anna Syreeni, Emma H. Dahlström, Bridget M. Lin, Nora Franceschini, Andrzej S. Krolewski, Valma Harjutsalo, Per-Henrik Groop, Niina Sandholm, on behalf of the FinnDiane Study Group

**Affiliations:** 1 Folkhälsan Institute of Genetics, Folkhälsan Research Center, Helsinki, Finland; 2 Department of Nephrology, University of Helsinki and Helsinki University Hospital, Helsinki, Finland; 3 Research Program for Clinical and Molecular Metabolism, Faculty of Medicine, University of Helsinki, Helsinki, Finland; 4 Department of Epidemiology, University of North Carolina, Chapel Hill, NC USA; 5 Section on Genetics and Epidemiology, Research Division, Joslin Diabetes Center, Boston, MA USA; 6 Department of Medicine, Harvard Medical School, Boston, MA USA; 7 Department of Diabetes, Central Clinical School, Monash University, Melbourne, Victoria Australia

**Keywords:** Diabetic kidney disease, METTL4, TNF receptors, TNFβ, Type 1 diabetes, Whole-exome sequencing, Whole-genome sequencing

## Abstract

**Aims/hypothesis:**

Diabetic kidney disease (DKD) is a severe diabetic complication that affects one third of individuals with type 1 diabetes. Although several genes and common variants have been shown to be associated with DKD, much of the predicted inheritance remains unexplained. Here, we performed next-generation sequencing to assess whether low-frequency variants, extending to a minor allele frequency (MAF) ≤10% (single or aggregated) contribute to the missing heritability in DKD.

**Methods:**

We performed whole-exome sequencing (WES) of 498 individuals and whole-genome sequencing (WGS) of 599 individuals with type 1 diabetes. After quality control, next-generation sequencing data were available for a total of 1064 individuals, of whom 541 had developed either severe albuminuria or end-stage kidney disease, and 523 had retained normal albumin excretion despite a long duration of type 1 diabetes. Single-variant and gene-aggregate tests for protein-altering variants (PAV) and protein-truncating variants (PTV) were performed separately for WES and WGS data and combined in a meta-analysis. We also performed genome-wide aggregate analyses on genomic windows (sliding window), promoters and enhancers using the WGS dataset.

**Results:**

In the single-variant meta-analysis, no variant reached genome-wide significance, but a suggestively associated common *THAP7* rs369250 variant (*p*=1.50 × 10^−5^, MAF=49%) was replicated in the FinnGen general population genome-wide association study (GWAS) data for chronic kidney disease and DKD phenotypes. The gene-aggregate meta-analysis provided suggestive evidence (*p*<4.0 × 10^−4^) at four genes for DKD, of which *NAT16 (*MAF_PAV_≤10%) and *LTA* (also known as *TNFβ*, MAF_PAV_≤5%) are replicated in the FinnGen general population GWAS data. The *LTA* rs2229092 C allele was associated with significantly lower TNFR1, TNFR2 and TNFR3 serum levels in a subset of FinnDiane participants. Of the intergenic regions suggestively associated with DKD, the enhancer on chromosome 18q12.3 (*p*=3.94 × 10^−5^, MAF_variants_≤5%) showed interaction with the *METTL4* gene; the lead variant was replicated, and predicted to alter binding of the MafB transcription factor.

**Conclusions/interpretation:**

Our sequencing-based meta-analysis revealed multiple genes, variants and regulatory regions that were suggestively associated with DKD. However, as no variant or gene reached genome-wide significance, further studies are needed to validate the findings.

**Graphical Abstract:**

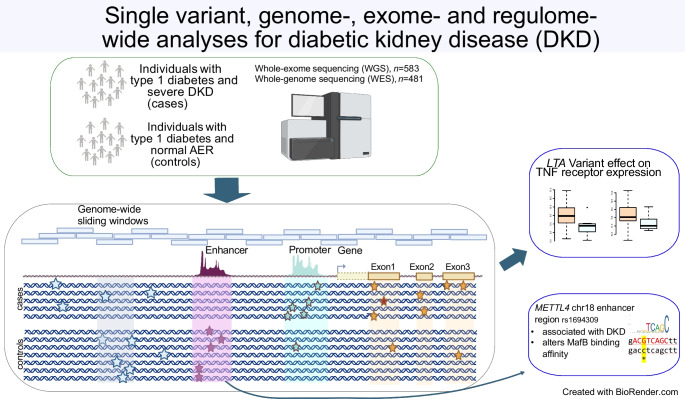

**Supplementary Information:**

The online version contains peer-reviewed but unedited supplementary material available at 10.1007/s00125-024-06241-1.


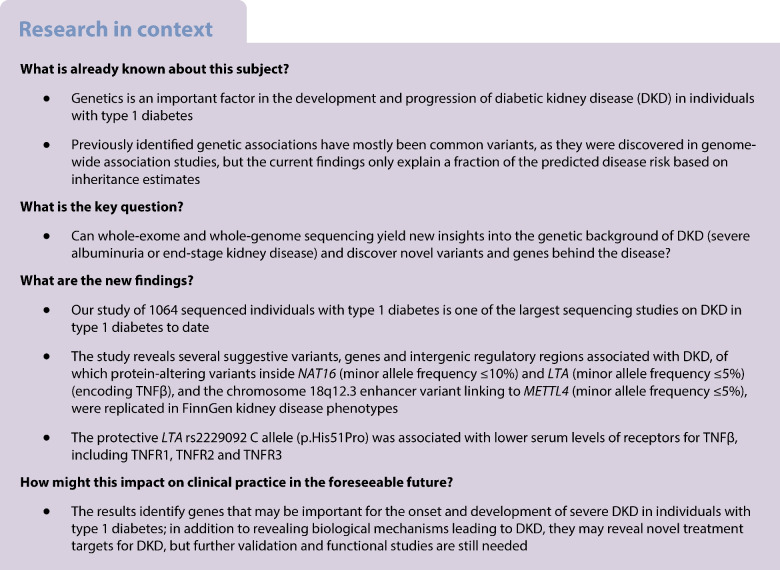


## Introduction

Type 1 diabetes is an autoimmune disease that is caused by destruction of the insulin-secreting beta cells in the islets of Langerhans in the pancreas. Long-term insulin irregularity leads to complications in several organs for a large proportion of individuals with type 1 diabetes [1]. In particular, prolonged hyperglycaemia leads to a decline of kidney function and diabetic kidney disease (DKD) in approximately 30% of individuals with type 1 diabetes [1, 2]. In the Western world, DKD is the most common cause of end-stage kidney disease (ESKD), which can be treated only through dialysis or kidney transplantation [3]. In addition, DKD predisposes the individuals to cardiovascular disease, and even early-stage DKD (moderate albuminuria) elevates the risk of myocardial infarction and stroke two- to threefold [4, 5].

Both genetic and environmental factors affect the occurrence of type 1 diabetes and its complications. Heritability estimates from genome-wide association studies (GWAS) suggest that genetic factors explain approximately one third of the DKD risk [6, 7]. The microarray-based chips used in GWAS include hundreds of thousands of common variable loci, and are thus excellent for the study of common variants that have modest effects on the disease risk [8, 9]. GWAS have shed light upon DKD mechanisms, but these earlier findings explain only a minority of the predicted genetic risk for DKD [10–13]. Our recent family-based linkage study and multi-cohort GWAS study suggested a role for rare genetic variants as risk factors for the development of DKD as well [14, 15]. Recently, whole-exome and whole-genome sequencing (WES and WGS, respectively) have enabled the study of low-frequency and rare variants that are expected to have larger effects on the disease risk. Although such rare signals are harder to detect, such studies have offered important information on complex trait and disease mechanisms, and led to drug discoveries [16]. Indeed, even though the rare variants do not contribute much to the total heritability of DKD, they provide clues to the mechanisms of DKD.

**Fig. 1:**
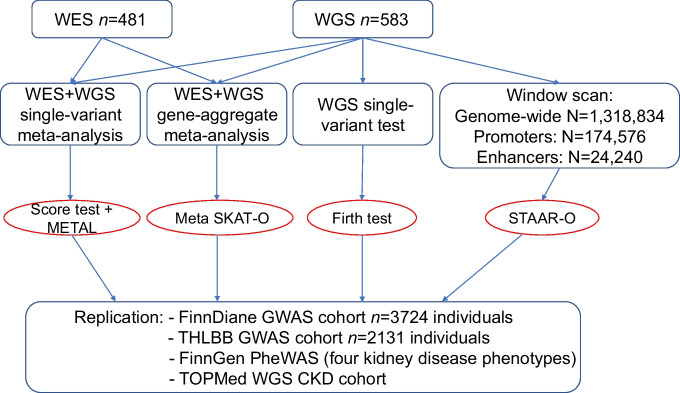
Study setup for single-variant and gene and intergenic region aggregate analyses. We performed single-variant and gene-aggregate meta-analyses on the WES and WGS cohorts, covering the exome regions and surrounding regions. The gene-aggregate analysis was run only for low-frequency PAVs and PTVs. We also performed a single-variant analysis and genome- and regulome-wide window scans using only WGS data. We replicated the results against FinnDiane and THL Biobank GWAS data, publicly available FinnGen data and TOPMed WGS data

WES offers a computationally simpler way of studying protein-altering variants (PAV) or protein-truncating variants (PTV) [17]. WGS studies additionally enable exploration of the intronic and intergenic regions, which may affect gene expression levels through transcription factor binding site activity or other regulatory processes [18]. Recently, a WGS study in mainly non-diabetic individuals from multiple ancestries identified three novel loci for eGFR [19]. However, there are currently only a few WES- or WGS-based studies for DKD. Our previous WES study on DKD in type 1 diabetes yielded no significant findings, whereas a recent WES study identified four exome-wide significant loci for DKD [6, 20]. Furthermore, our previous WGS study of 74 sibling pairs proposed involvement of protein kinase C family members in DKD [14, 18]. Here, we use WES and WGS meta-analyses to study the genetic background of DKD. As we had limited power to detect low-frequency single-variant associations, we performed gene-aggregate tests as well as genome-wide and regulome-wide scans for the non-coding regions using the WGS data (Fig. 1).

## Methods

### Study participants

All participants were recruited from the Finnish Diabetic Nephropathy Study (FinnDiane). FinnDiane is an ongoing nationwide prospective study that was established in 1997 to pinpoint risk factors for long-term diabetic complications. All Finnish individuals with type 1 diabetes were invited during the active recruitment period, and the study currently comprises more than 6000 individuals with type 1 diabetes, representing over 10% of individuals with type 1 diabetes in Finland. The sex of the participants was assigned by national registry data, based on genetic data. The study protocol was approved by the Ethical Committee of the Helsinki and Uusimaa Hospital District (491/E5/2006, 238/13/03/00/2015 and HUS-3313–2018, 3 July 2019), and participants gave their informed consent before recruitment. This study was performed according to the principles of the Declaration of Helsinki. In brief, data on diabetic complications, history of cardiovascular events and prescribed medications were registered using standardised questionnaires, and blood and urine samples were collected during a standard visit to the attending physician. Participants were diagnosed with type 1 diabetes by their attending physician, had an onset of diabetes before the age of 40 years, and started permanent insulin treatment within the first year after the diagnosis [21]. DKD was defined based on albuminuria status, and participants with DKD had either severe albuminuria (≥200 μg/min) in two of three consecutive urine collections, or ESKD requiring dialysis or a kidney transplant, whereas control participants had a normal AER (AER <20 μg/min or equivalent) throughout the follow-up period.

### Sequencing and data analysis

WES and WGS were performed for 498 and 599 individuals, respectively. WES was performed at the University of Oxford, UK, using an Illumina HiSeq2000 platform (San Diego, USA), with an average requirement of 20× target capture with ≥80% coverage, as described previously [6]. WGS was performed by Macrogen (Rockville, MD, USA) using an Illumina HiSeq X platform, with a requirement of >30× average coverage for mapped reads. Based on initial quality control performed at the University of Oxford for WES, or by Macrogen for WGS, 27 WES samples and 15 WGS samples were excluded due to a high homozygosity/heterozygosity ratio, abnormally low mapping depth or mapped PCR reads.

The WES and WGS samples were processed using the Genome Analysis Toolkit (GATK) version 4 golden standard pipeline, and annotated using SnpEff version 5.0e [22, 23]. First, the fastq reads were trimmed using Trimmomatic version 0.36, the trimmed reads were run through FastQC version 0.11.9, and the results were aggregated and assessed using MultiQC version 1.11. The reads were aligned by lanes (1–8 lanes for WGS and 1 or 2 lanes for WES) and sorted, and duplicates were removed using Picard’s SortSam and MarkDuplicates tools. The reads were recalibrated by chromosome using the GATK BQSR and ApplyBQSR tools, and variants were called using the ERC mode of HaplotypeCaller tool into a single- sample GVCF file. The GVCF files were combined into a multi-sample GVCF files using the GATK CombineGVCFs tool, and transformed into a VCF file using the GATK GenotypeGVCFs tool, separately for WES and WGS samples. Variants were then filtered using an excess heterozygosity threshold of 54.69. SNPs and indels were filtered separately according to tranche thresholds recommended by GATK, with a truth sensitivity level of 99.7%. All variants were annotated using SnpEff version 5.0e based on the GRCh38.99 database (ftp.ensembl.org/pub/release-99/gtf/homo_sapiens). Comparison with pre-existing GWAS genotyping [24] showed 99.5% concordance with the sequencing data. To prepare chromosome 6 with ALT contigs (HLA region), we used a workflow from https://gatk.broadinstitute.org/hc/en-us/articles/360037498992--How-to-Map-reads-to-a-reference-with-alternate-contigs-like-GRCH38/ with modifications for GATK4. Post-pipeline variants with <98% call rate and a Hardy–Weinberg Equilibrium *p* value <1 × 10^−10^ were excluded. The relatively lenient *p* value threshold was selected because all participants had type 1 diabetes, and therefore all type 1 diabetes-associated variants are expected to show some deviation from Hardy–Weinberg equilibrium. Genetic principal components (PCs) were calculated using PLINK 1.9 (http://pngu.mgh.harvard.edu/~purcell/plink/) [25], and visual inspection of the first two PCs indicated no population outliers (Electronic Supplementary Material [ESM] Fig. 1). These first two PCs were used as covariates in all main analyses.

### Variant-based tests

We performed single-variant genome-wide association testing for the WGS data with Firth regression, using age of diabetes onset, sex and the two first genetic PCs as covariates. For meta-analysis of the WES and WGS data, we performed association testing with score test and meta-analysis using the inverse variant-weighted method. In WGS single-variant analysis, variants with a *p* value <5 × 10^−8^ were considered genome-wide significant, and those with a *p* value <5 × 10^−5^ were considered suggestive. In the WES/WGS meta-analysis, a *p* value <3.5 × 10^−7^ was considered to indicate exome-wide significance, and a *p* value <3.5 × 10^−5^ was considered suggestive. All single variants were considered without a minor allele frequency (MAF) filter. Single-variant tests were performed using RVTESTS (version 2.1.0) and the single-variant meta-analysis was performed using METAL version 2011–03–25 [26, 27]. Power calculations were performed using the ‘genpwr’ package in R v4.2.1 (R Foundation for Statistical Computing, Austria), based on the sample numbers in the combined WES and WGS data.

### Gene aggregation tests

We performed gene-based tests on WES and WGS data separately for PTVs (including frameshift and nonsense variants, and loss or gain of start or stop codons) and PAVs (including missense variants, in-frame insertions and deletions [INDELS] and PTVs) (ESM Table 1), assigned to specific genes within RefSeq exomes. We performed six separate gene-aggregate analyses based on PAVs and PTVs using MAF filters of ≤1% (rare), ≤5% (low frequency) and ≤10% (extended uncommon). *p* values <4 × 10^−6^ were considered significant (adjusted for 18,226 genes with PAVs with an MAF ≤10%) and *p* values <4 × 10^−4^ were considered suggestive. Gene-based SKAT-O meta-analysis was performed for WES and WGS cohorts using the MetaSKAT version 0.8.1 R package [28]. Results were filtered by including only genes with a cumulative minor allele count (MAC) ≥5 across all included variants within each gene.

### Look-up of monogenic kidney disease genes

We studied whether known monogenic kidney disease genes were significantly enriched in our gene-based meta-analysis for DKD. Altogether, we considered 464 unique genes causing syndromic or monogenic kidney diseases as listed by Connaughton et al [29].

### Whole-genome sliding-window and regulome-wide analysis

We performed genome-wide sliding-window tests and aggregation tests for promoters and enhancers for WGS data using the omnibus STAAR-O test in the STAAR version 0.9.6 R package [30]. The genome-wide window scan tests were performed using partly overlapping windows with a window size of 4000 bp, with a shift of 2000 bp between the window start sites, and a minimum of five variants within the region. Importantly, sliding-window analyses were performed by weighting the variants using Combined Annotation Dependent Depletion (CADD) version 1.6 functional annotations, variant rarity and annotation PCs, as calculated and implemented previously [30, 31]. We used FANTOM5 atlas cap analysis of gene expression (CAGE) profiles for both promoter and enhancer regions [32, 33], measured across multiple human primary cell lines, tissues and cancer cell lines, and converted from GRCh37 to GRCh38. The CAGE transcription start sites were extended to form full-length promoters (1000 bp), while variants within enhancer and promoters were weighted only according to variant rarity (a functional annotation that is available for all variants). At least two variants were required for these regulatory region tests. An MAF threshold of ≤5% was used for all tests, and a cumulative MAC ≥5 was required for all regions. Altogether 184,609 promoters and 24,240 enhancers were tested, resulting in Bonferroni-corrected significance levels of *p*<2.86 ×  10^−7^ and 2.12 × 10^−6^.

We aimed to identify putative enhancer target genes using Zenbu promoter capture Hi-C data from the YUE Lab database (http://3dgenome.fsm.northwestern.edu/chic.php) [34]. As no Hi-C data were available for kidneys, we searched for bladder as a related tissue.

### Replication of findings in the FinnDiane GWAS

Variants and genes were replicated against FinnDiane GWAS data [24]. Genomes were genotyped in four batches at the University of Virginia using HumanCoreExomBead arrays 12-1.0, 12-1.1 and 24-1.0 (Illumina, USA). The GWAS data were converted from GRCh37 to GRCh38 genetic coordinates [35]. We removed individuals with high genotype missingness (>5%) and variants with excess heterozygosity (± 4 SD), high missingness (>2%), a low Hardy–Weinberg equilibrium *p* value (<10^−6^) or an MAC <3. For imputation, genotypes were pre-phased using Eagle 2.3.5 and imputed using Beagle 4.1 (version 08Jun17.d8b), based on the population-specific SISu version 3 reference panel consisting of WGS data for 3775 Finnish individuals. Variants were annotated using SnpEff version 5.0e [22]. Altogether, 6449 individuals and 15.21 million variants with imputation quality *r*^2^>0.7 passed the quality control. After exclusion of individuals included in the WES or WGS studies, the replication was tested in 3724 individuals with type 1 diabetes, of whom cases had severe albuminuria or ESKD, and control individuals had normal AER with a minimum diabetes duration of 26 years.

Additionally, replication was attempted in GWAS data for 2356 non-overlapping Finnish individuals with type 1 diabetes from the THL Biobank diabetes studies collection (thl.fi/biobank), with registry data for ESKD based on ICD-10 codes (https://icd.who.int/browse10/2019/en) being available for 2131 individuals. These GWAS data were imputed using the same pipeline as the main data, with the SISu version 4 reference panel, and included 16.94 million variants after the quality control. As albuminuria data were not available for these individuals, they were grouped based on ESKD occurrence: there were 70 individuals with ESKD, and 2061 individuals without ESKD. Single-variant replications were conducted using the Firth test with RVTESTS and gene-aggregate replications were conducted using the SKAT-O test with REGENIE [36].

Single-variant replication was further tested using Finnish general population GWAS data from the FinnGen project r9 release (https://r9.finngen.fi/) covering four kidney disease phenotypes. Finally, we replicated the three non-HLA region genes and 16 SNPs against the Trans-Omic for Precision Medicine (TOPMed) eGFR WGS study on multi-ancestry general population with 23,732 individuals [19]. *p* values <0.0029 were considered Bonferroni significant after correction for 17 tested variants.

### In silico annotation of the lead genes and variants

We queried the Human Kidney Expression Quantitative Trait Locus (eQTL) Atlas to identify the variants associated with gene expression levels [37]. Differential gene expression was investigated for kidney condition datasets in the Nephroseq classic portal version 4. The Transcription Factor Affinity Prediction (TRAP) tool (http://trap.molgen.mpg.de/cgi-bin/trap_two_seq_form.cgi) was used to predict transcription factors with differential binding affinity to the lead regulatory variant reference and alternative allele sequences by searching the JASPAR and TRANSFAC vertebrate motifs with the human background model [38–40]. Linkage disequilibrium was evaluated using NIH LDlink tools (https://ldlink.nih.gov/) in the Finnish population.

### Measurement of TNF receptor levels and HLA haplotypes

Serum levels of 21 proteins previously shown to be associated with DKD progression and/or ESKD development, including three TNF receptors (TNFRSF1A/TNFR1, TNFRSF1B/TNFR2 and TNFRSF3/TNFR3) that bind lymphotoxin-α (LT-α), were measured at Olink Proteomics (Uppsala, Sweden), using an Olink Proseek Multiplex proteomic platform with proximity extension assay method. The protein concentrations were pre-processed, log_2_-transformed and normalised into ‘normalized protein expression values’ (NPX). Measurements were performed for 740 individuals; WES/WGS data were available for 128 of these individuals. Of these, eight had at least one rs2229092 alternative allele (seven had the C/A genotype, one had the C/C genotype). Variant association with the circulatory biomarkers was tested using linear regression in R, and the analyses were adjusted for age and sex. All 128 participants had severe albuminuria at the time of sample collection due to the Olink study design (A. S. Krolewski, unpublished data).

HLA alleles were previously imputed based on GWAS data and were available for 1021 individuals [14]. Based on a previously published OR table [41], we considered the HLA genotype as high risk if the individual had one high-risk haplotype (OR≥3.64) combined with one moderate risk or neutral haplotype (OR≥0.87).

## Results

In both the WES and WGS cohorts, the cases and control individuals had a similar age of type 1 diabetes onset and similar BMI, but the control individuals had longer duration of diabetes, and, based on the study design, higher baseline eGFR (Table 1). There was also a higher proportion of women among control individuals compared with cases. All the following analyses were adjusted for sex, age of diabetes onset, and the first two PCs of the genetic PC analysis.

**Table 1:** Clinical characteristics of participants with DKD or normal AER in the WES and WGS cohorts

	WGS			WES		
	DKD	Normal AER	*p* value	DKD	Normal AER	*p* value
*n*	291	292		250	231	
DKD class						
Severe albuminuria	16			125		
ESKD	275			125		
Sex (% female)	28.9	60.6	<0.001	47.5	61.8	<0.001
Age at diabetes onset (years)	11.8 (6.8, 17.0)	12.3 (7.3, 16.9)	NS	12.6 (6.8, 17.0)	12.1 (7.7, 18.0)	NS
Duration of diabetes (years)	29 (25, 35)	40 (38, 45)	<0.001	24 (20, 33)	43 (40, 48)	<0.001
eGFR (ml/min per 1.73m^2^)	10 (10, 10)	94 (83, 102)	<0.001	10 (10, 50)	89 (77, 99)	<0.001
HbA_1c_ (%)	9.0 (8.0, 9.9)	8.0 (7.5, 8.4)	<0.001	8.6 (7.7, 9.9)	8.3 (7.7, 8.9)	NS
HbA_1c_ (mmol/mol)	75 (64, 85)	64 (58, 68)	<0.001	70 (61, 85)	67 (61, 74)	NS
BMI (kg/m^2^)	23.8 (21.2, 27.8)	24.5 (22.8, 26.5)	NS	25.1 (21.9, 27.1)	25.1 (22.9, 27.9)	NS
Systolic BP	148 (134, 165)	135 (123, 145)	<0.001	141 (129, 154)	138 (128, 149)	NS
Diastolic BP	84 (76, 92)	77 (70, 82)	<0.001	85 (79, 92)	76 (71, 82)	<0.001
Total cholesterol (mmol/l)	5.23 (4.36, 6.00)	4.74 (4.22, 5.26)	<0.001	4.89 (4.47, 5.54)	5.22 (4.66, 5.88)	0.001
Triacylglycerol (mmol/l)	1.56 (1.10, 2.28)	0.83 (0.64, 1.06)	<0.001	0.87 (0.72, 1.14)	1.34 (0.99, 2.04)	<0.001
Smokers	65.6	40.8	<0.001	38.4	55.4	<0.001

Values are *n* or % for categorical variables and median (IQR) for continuous variables. Characteristics are those at the timepoint of DKD classification used in the analysis (i.e. most severe observed phenotype by the time of analysis) or the timepoint closest to that date based on available longitudinal data (HbA_1c_, BMI, systolic and diastolic BP). Smoking status (current or former smoker vs never smoker) and lipid values were recorded at the study baseline

### Single-variant associations

**Fig. 2:**
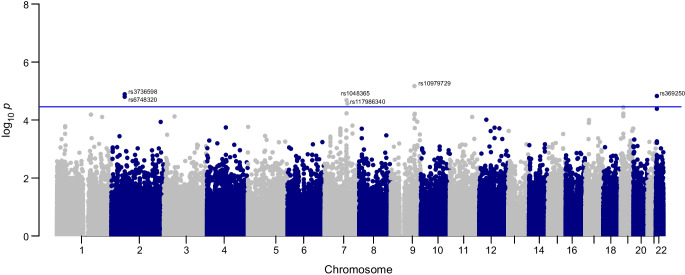
Manhattan plot for the WES/WGS single-variant meta-analysis. The meta-analysis resulted in identification of six variants: five common ones (chr2 *FAM161A* rs3736598 [*p*=1.30 × 10^−5^], rs6748320 [*p*=1.58 × 10^−5^], chr22 *THAP7* rs369250 [*p*=1.50 × 10^−5^], chr7 *AP1S1* rs1048365 [*p*=2.07 × 10^−5^] and *KMT2E* rs117986340 [*p*=2.71 × 10^−5^]), and one with a low frequency (chr9 *EPB41L4B* rs10979729 [*p*=6.76 × 10^−6^])

The meta-analysis of WES and WGS data identified six variants, five common and one with low frequency, including two PAVs, with a suggestive *p* value of 3.5 × 10^−5^ (Table 2 and Fig. 2). The experiment had 80% power to detect low-frequency variants with an MAF of 5% and an OR=4.0 with genome-wide significance (α<5 × 10^−8^) or an OR=2.8 with suggestive significance (α=3.5 × 10^−5^; ESM Fig. 2). Extending the analysis to non-coding regions, the single-variant association test for WGS data identified variants on intergenic regions 14p12 (close to *CPSF2*) and 16p11.1 (near a group of RNA genes and pseudogenes) and two intronic variants in *MYO9B* that were suggestively associated with DKD (*p*<5 × 10^−6^; ESM Table 2).

**Table 2:** Suggestive associations (*p*<3.5 × 10^−5^) in the WES/WGS single-variant-based meta-analysis for DKD (*n*=1064)

Position^a^	Variant	A1/A2^b^	Gene	Annotation	MAF	*p* value^c^ (WGS/WES)	OR (95% CI)^d^	*p* value^e^
9:109,176,646	rs10979729	T/C	*EPB41L4B*	C→T (Pro846Pro)	0.07	1/0.3385	2.09 (1.53, 2.87)	6.76 × 10^−6^
2:61,825,245	rs3736598	A/G	*FAM161A*	Non-coding	0.40	0.55/0.58	0.67 (0.56, 0.8)	1.30 × 10^−5^
22:21,002,277	rs369250	G/A	*THAP7*	Promoter–TSS	0.49	0.047/0.24	1.42 (1.24, 1.77)	1.50 × 10^−5^
2:61,826,155	rs6748320	A/G	*FAM161A*	Non-coding	0.40	0.55/0.64	0.67 (0.56, 0.8)	1.58 × 10^−5^
7:101,1611,49	rs1048365	T/C	*AP1S1*	3′ UTR	0.12	0.87/0.32	0.58 (0.45, 0.74)	2.07 × 10^−5^
7:105,107,452	rs117986340	G/T	*KMT2E*	G→T (Gly999Cys)	0.14	0.57/0.66	1.81 (1.37, 2.39)	2.71 × 10^−5^

^a^Chromosomal position given as chromosome:base pairs using the GRCh38 genome build

^b^Major/minor alleles

^c^*p* value for Hardy–Weinberg equilibrium in the WGS/WES data

^d^OR and 95% CI for the minor allele

^e^*p* value for association with DKD

TSS, transcription start site; UTR, untranslated region

We tested for replication of the suggestive single-variant DKD associations in the non-overlapping FinnDiane and THL Biobank GWAS data for individuals with type 1 diabetes (ESM Table 3), in the general population FinnGen GWAS data using four kidney disease definitions (ESM Table 4), and in the TOPMed WGS data for chronic kidney disease (CKD). The *THAP7* promoter rs369250 was replicated for CKD (*p*=2.7 × 10^−4^, Bonferroni significant) and DKD (*p*=0.012) in the FinnGen GWAS data (ESM Table 4). The variant showed significant eQTL activity in human kidneys with *THAP7-AS1* (*p*=8.488 × 10^−45^) (ESM Table 5).

### Gene-aggregate tests

In the gene-aggregate meta-analysis, *LTA* and *TSEN54* reached a suggestive *p* value <4.0 × 10^−4^ for analyses with low-frequency or rare PAVs (Table 3). In addition, *NAT16* and *SLC10A6* reached the same suggestive threshold when analysis was extended to variants with an MAF ≤10%*. NAT16* contained six PAVs with an MAF ≤10%, and the association was driven by rs34985488, which is associated with higher odds of DKD (A→C p.Phe63Cys, *p*=5.82 × 10^−5^; ESM Table 6). The variant was predicted to be deleterious or possibly damaging by the SIFT [42] and PolyPhen [43] algorithms, respectively. On the HLA region, association at *LTA* was based on only one PAV, p.His51Pro (rs2229092), which was associated with lower risk of DKD (*p*=1.5 × 10^−4^).

**Table 3:** Genes with a suggestive *p* value of <4 × 10^−4^ in the SKAT-O gene-aggregate meta-analysis (*n*=1064)

Gene	Position	*p* value	MAF threshold	Variants tested	Variant type	Replication^a^	Previous findings
*NAT16*	7:101,170,496–101,180,293	1.4 × 10^−4^	0.1	6	PAV + PTV	*NAT16* rs34985488 FinnGen *p*_CKD_=0.0028^b^	Significant difference in gene expression between ING patients and control individuals [45]
*LTA*^c^	6:31,560,610–31,574,324	1.5 × 10^−4^	0.05	1	PAV + PTV	*LTA* rs2229092 FinnGen *p*_T1D_with_kidney_complications_=0.0044	DKD candidate gene [48]
*SLC10A6*	4:86,823,468–86,849,384	2.7 × 10^−4^	0.1	3	PAV + PTV		
*TSEN54*	17:75,515,944–75,524,735	3.7 × 10^−4^	0.01	14	PAV + PTV		*TSEN2* splice site mutation associated with atypical haemolytic uraemic syndrome [53]

^a^Replication results with *p* value <0.05 are shown

^b^*p* values <0.0029 were significant after Bonferroni correction for 17 tested variants

^c^*LTA* is localised on the HLA region, for which a different variant-calling pipeline was used (see Methods for further details)

ING, idiopathic nodular glomerulosclerosis

We sought replication of these four genes in FinnDiane GWAS, THL Biobank GWAS, TOPMed WGS and UK Biobank WES gene-aggregate data. Furthermore, for the single variants within these genes that were nominally significant (*p*<0.05) in the FinnDiane WES/WGS meta-analysis, we tested for replication in the FinnDiane, THL Biobank, FinnGen GWAS and TOPMed WGS data. No replication was observed in the gene-aggregate tests (ESM Table 7), but *NAT16* rs34985488 was replicated in the FinnGen GWAS for the CKD phenotype (*p*=0.0028; Bonferroni significant) and *LTA* rs2229092 was replicated for the type 1 diabetes with kidney complications phenotype (*p*=0.0044, ESM Table 4). *LTA* rs2229092 was also associated with a lower risk of type 1 diabetes (OR 0.74, *p*=3.4 × 10^−16^), possibly reflecting linkage disequilibrium with classical type 1 diabetes HLA DR/DQ haplotypes. We therefore tested whether the observed DKD association could be explained by HLA DR/DQ alleles and genotypes or a more severe diabetes phenotype. Altogether, 55% of the individuals had a high type 1 diabetes risk HLA genotype (either high/high or high/neutral haplotypes, ESM Table 8). The *LTA* rs2229092 remained associated with DKD when the high-risk HLA genotype was included as a covariate in the model (*p*=5.2 × 10^−4^), whereas the HLA risk genotype was not associated with DKD (*p*=0.13). These results support the inference that the rs2229092 signal is separate from the classical HLA DR/DQ type 1 diabetes associations. It has previously been established that type 1 diabetes risk HLA haplotypes are associated with low random serum C-peptide concentrations and younger age at type 1 diabetes onset, and therefore we also tested whether rs2229092 is associated with these proxies [44]. The variant was not associated with age of type 1 diabetes onset (14.8 vs 13.2 years, *p*=0.07) or C-peptide levels at study baseline (0.009 vs 0.015 nmol/l, *p*=0.44), supporting its association with DKD rather than type 1 diabetes in our data.

**Fig. 3:**
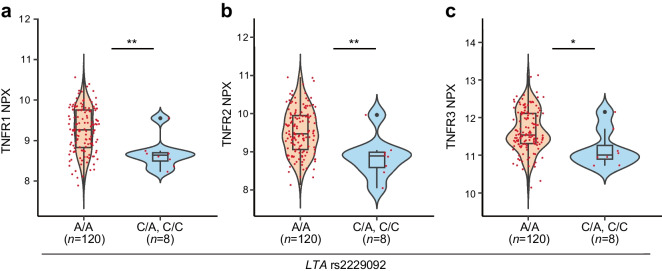
Association of the *LTA* rs2229092 C allele and lower levels of TNF receptors. Serum levels of three TNF receptors that bind LT-α (TNFR1, TNFR2 and TNFR3) were measured using the Olink platform, and serum TNF receptor levels were measured for 128 individuals with WES and WGS data. Linear regression was adjusted for age and sex. The alternative C allele of *LTA* rs2229092 had significantly lower levels of (**a**) TNFR1, (**b**) TNFR2 and (**c**) TNFR3 compared with carriers of the reference allele. Differences between the groups are indicated by asterisks: *p<0.05, **p<0.01

*LTA* encodes LT-α, also known as TNFβ, which binds to TNF receptors. We took a subset of 128 FinnDiane participants for whom targeted Olink proteomics data were also available. The reference and alternative allele carriers had similar clinical characteristics (ESM Table 9), and the alternative C allele of *LTA* rs2229092 was associated with lower levels of serum TNF receptors, including TNFR1 (*p*=0.005), TNFR2 (*p*=0.003) and TNFR3 (*p*=0.017) (Fig. 3).

### Enrichment of monogenic or syndromic kidney disease genes

We further tested whether monogenic kidney disease genes are enriched among DKD-associated genes (ESM Table 10). The strongest evidence of enrichment occurred among the ‘cystic kidney disease or nephronophthisis’ class, as 11 of 96 of the monogenic genes (11%) were associated (*p*<0.05) with DKD in our WES/WGS meta-analysis (binomial test, *p*=0.004).

### Intergenic variant aggregate tests

To improve the statistical power to discover non-coding genetic factors behind DKD, we performed promoter and enhancer aggregate analyses and functionally informed genome-wide sliding-window analyses. Two enhancers located at 18q12.3 (*p*=6.78 × 10^−5^) and 9q21.11 (*p*=2.17 × 10^−4^) were suggestively associated with DKD (Table 4). Based on Hi-C data, the chromosome 18q12.3 region showed enhancer activity with the closest protein-coding gene *METTL4* (located 186 kb upstream) in the bladder. The 9q21.11 locus, close to *CTSL*, is a promoter–enhancer region, and showed enhancer activity with *CTSL* (ESM Table 5).

**Table 4:** STAAR-O aggregate analysis results for genome-wide sliding windows (*n*=1,318,834 regions), promoters (*n*=184,609) and enhancers (*n*=24,240); the analysis was performed for the WGS dataset only (*n*=583)

Region	Variant count	Cumulative MAC^a^	*p* value	MAF threshold	Closest gene
Sliding windows					
12:108,104,001–108,108,000 (12q14.3)	14	22	4.66 × 10^−6^	0.01	*WSCD2*
4:75,122,001–75,126,000 (4q13.3)	21	61	9.46 × 10^−6^	0.01	
Promoters					
9:87,792,126–87,793,126 (9q21.11)	6	79	2.67 × 10^−5^	0.05	*CTSL3P* (pseudogene)
2:40,755,215–40,756,215 (2q14.2)	5	14	3.17 × 10^−5^	0.05, 0.01	*LINC01794* (lncRNA)
Enhancers					
18:2,350,865–2,351,244 (18q12.3)	2	24	3.94 × 10^−5^	0.05	*METTL4*; *MYOH1*
9:87,792,785–87,793,201 (9q21.11)	4	27	1.17 × 10^−4^	0.05	*CTSL*

^a^Cumulative minor allele count (number of copies of the minor allele) across all the included variants within the given MAF class

In the sliding-window analysis, the strongest associations were obtained for a chromosomal window on 12q14.3, 21 kbp upstream of *WSCD2* (*p*=4.66 × 10^−6^) and intergenic region 4q22.3 (*p*=9.46 × 10^−6^). However, none of the genomic windows (1,318,834 regions), promoter regions (184,609 regions) or enhancer regions (24,240 regions) remained significant after Bonferroni correction (Table 4).

Replication for the genome-wide sliding-window, promoter- and enhancer-wide analyses was performed by testing the nominally significant variants (ESM Table 11) in the replication data. We observed replication for rs183413211 of the 2q14.2 region 106 kb from *SLC8A1* (TOPMed *p*_CKD_=0.042; ESM Table 3) and rs16943099 in the *METTL4* enhancer region in 18q12.3 (FinnGen *p*_T2D_with_renal_complications_=8.6 × 10^−4^ [Bonferroni significant] and *p*_DKD_=0.036; ESM Table 4).

To assess the potential functional effect of the variants within the identified enhancer regions, we applied the TRAP prediction tool to estimate whether the variants affect the transcription factor binding probability of the sequence. For the *METTL4*/18q12.3 enhancer region lead SNP, rs16943099, only the reference allele was predicted to provide a binding site for podocyte-specific transcription factor MafB (TRANSFAC V$MAFB_01, *p*_REF_<1.75 × 10^−6^), which is absent in the alternative allele sequence (*p*_ALT_=0.239; ESM Fig. 3 and ESM Table 5).

### Sub-analyses of the lead variants with DKD phenotypes and covariates

We performed additional phenotypic analyses to further characterise the 17 lead variants from single-variant, gene-aggregate, sliding-window and regulome analyses. Additional adjustment one by one for BMI, diastolic BP, systolic BP, HbA_1c_, smoking status, total cholesterol and triacylglycerol levels generally modestly reduced the associations with DKD, but they remained significant (ESM Table 12). Furthermore, all lead variants showed highly similar or slightly lower associations when only individuals with ESKD (*n*=939) were included in the analysis (ESM Table 13). Finally, as some previous DKD associations have been shown to be sex-specific, we additionally tested for variant–sex interaction, and rs16943099 adjacent to *METTL4* showed significant interaction with sex (*p*=0.01), with a significant association detected in men (OR 0.55; 95% CI 0.43, 0.69; *p*=1.3 × 10^−6^) but not in women (OR 0.88; 95% CI 0.66, 1.18; *p*=0.402) (ESM Table 13 and ESM Fig. 4).

## Discussion

We studied 1064 Finnish individuals with type 1 diabetes, representing the extreme phenotypes for DKD, to identify novel rare and low-frequency variants associated with DKD. We included 546 individuals with severe DKD (severe albuminuria or ESKD) and 528 with type 1 diabetes but normal AER despite a diabetes duration of at least 25 years. We performed genome-wide single-variant analyses, including an analysis of the HLA region, and used both gene and regulatory region aggregate tests to identify low-frequency variants, extending to an MAF ≤10%, associated with DKD susceptibility, revealing several putative associations with novel and functionally plausible genes for DKD.

The gene-aggregate meta-analysis for low-frequency and rare PAVs and PTVs identified two genes, *LTA* and *TSEN54*, as suggestively associated with DKD. As our previous GWAS also identified a common missense variant associated with DKD, namely the *COL4A3* missense variant rs55703767 [10], we further extended the analysis to more common variants with an MAF ≤10%, resulting in suggestive association at *NAT16* and *SLC10A6*. Through look-ups of the lead SNPs behind the gene-aggregate results, replication was found for *NAT16* and *LTA* in the FinnGen cohort (ESM Table 3). *NAT16* putatively encodes *N*-acetyltransferase 16. *N*-acetyltransferases transfer acetyl groups from acetyl-CoA to molecules such as arylamines. According to GTEx RNA-seq data (https://www.gtexportal.org/home/gene/NAT16), *NAT16* is expressed in the kidney cortex and medulla, along with most other tissues, and *NAT16* was previously found to show significantly higher expression in individuals with idiopathic nodular glomerulosclerosis compared with healthy control individuals (log_2_ fold change=7.11, *p*=1.67 × 10^−8^) [45]. The lead SNP rs34985488 was classified as deleterious by SIFT and was replicated in the FinnGen CKD phenotype dataset (*p*=0.0028). Interestingly, the *NAT16* missense variant rs34985488, and one of the single-variant lead SNPs, rs1048365 in the *AP1S1* 3′ untranslated region, are in moderate linkage disequilibrium (ESM Fig. 5).

The HLA region gene *LTA*, which encodes LT-α/TNFβ, plays an important role in the immune response, inflammation and apoptosis. LT-α binds to TNF receptors TNFR1 and TNFR2 in its homotrimeric form, and to TNFR3 in its heterotrimeric form with LT-β/TNFC; all three TNF receptors have been shown to be associated with progression of DKD [46, 47]. The *LTA* association with DKD was driven by rs2229092 (OR 0.39, *p*=1.47 × 10^−4^), and the association was replicated in the FinnGen type 1 diabetes with kidney complications dataset (*p*=0.0044). However, in the single-variant association meta-analysis, rs2229092 did not reach study-wide significance or the suggestive threshold, possibly due to lack of power. The alternative C allele of rs2229092 was associated with lower serum levels of multiple TNF receptors, including TNFR1, TNFR2 and TNFR3, suggesting that the variant protects from DKD through reduced inflammatory responses. The variant is not a known trans-eQTL for any of the TNF receptors, and we hypothesise that the association is due to a biological feedback loop between circulating TNFβ concentrations and its corresponding TNF receptors. Of note, an association between DKD and *LTA* p.T60N (rs1041981) was already suggested by candidate gene studies 15 years ago, but the association has not been replicated in more recent large GWAS [48]. This may be partly due to exclusion of the HLA region from GWAS imputation or sequencing due to the complexity of the region. Interestingly, in the FinnGen r9 dataset, rs2229092 was shown to confer protection against type 1 diabetes, other autoimmune diseases and diabetic complications, with ophthalmic complications being the most significant (OR 0.70, *p*=4.5 × 10^−13^; ESM Table 3). Other researchers have noted that assessing causal effects of rs2229092 is difficult due to pleiotropy [49]. However, adjusting the association for the HLA risk genotype did not affect the association with DKD, suggesting that the association detected here reflects DKD rather than type 1 diabetes risk.

The WGS data allowed us to investigate low-frequency variants also in non-coding regions. On chromosome 18q12.3, an enhancer interacting with the *METTL4* gene was suggestively associated with DKD (*p*=6.78 × 10^−5^). The association was led by rs16943099, and it replicated for ‘type 2 diabetes with kidney complications’ (*p*=8.6 × 10^−4^) in the FinnGen GWAS data. In silico prediction suggested that the rs16943099 minor C allele, which is associated with a lower risk of DKD, disrupted a transcription factor binding site for the podocyte-specific transcription factor MafB (ESM Fig. 3 and ESM Table 5); forced *mafb* expression was recently shown to prevent CKD in mice [50]. *METTL4* encodes Mettl4 methyltransferase, which has been shown to mediate m^6^Am methylation on U2 snRNA in vitro [51]. An intergenic variant rs185299109 in the *LINC00470*/*METTL4* locus was previously found to be associated with DKD (eGFR-based CKD phenotype, *p*=1.3 × 10^−8^) [10]. Moreover, methyl adenosine modification of the paralogous gene *METTL3* was observed to promote podocyte injury in DKD [52], and a rare intronic variant in *METTL8* was one of the novel findings for eGFR in the TOPMed WGS [19].

To our knowledge, our WES/WGS analysis of 1064 individuals with type 1 diabetes is one of the largest sequencing studies for DKD to date. However, previous studies have shown that a larger sample size is often needed to discover variants with modest effect size, and thus the main limitation of this study is the sample size. We only had limited statistical power to detect single variants associated with DKD, and the putative associations detected in the single-variant analyses should be interpreted with caution. The aggregate analyses increase the statistical power by increasing the number of carriers and reducing the burden of multiple testing. While gene-aggregate analyses are commonly used in exome sequencing studies, the functionally weighted regulatory region aggregate analyses of the WGS data provide a novel way to identify regulatory variant associations more robustly. Furthermore, the study participants were carefully selected and characterised for their phenotype, and had either advanced DKD (with the majority developing ESKD) or a long duration of type 1 diabetes without DKD. Of note, although the discovery cohort’s phenotype was based on severe albuminuria or ESKD, many of the variants replicated for the eGFR-based CKD definition or a more general definition of type 1 and 2 diabetes with renal complications. We were unable to replicate common variants previously identified for DKD, such as the reported *COL4A3* missense variant rs55703767 (*p*=0.056), suggesting that we may have missed variants that are relevant for DKD due to the limited sample size. However, our main focus was the discovery of low-frequency and rare variants with functional relevance. We did not perform the genome-wide analyses separately for men and women due to the limited power, but, of the tested variants, the METTL4-adjacent rs16943099 showed significant interaction with sex, and associated with DKD only in men.

We used two different sequencing platforms in the study. Even though the datasets were analysed using the same pipeline, there were differing read lengths of 150 bp for WGS and 100 bp for WES, and the mean number of low-frequency variants per gene was significantly greater in the WGS study (5.88) compared with the WES dataset (5.31). Due to the limitation of databases and tools, our study included only transcribed enhancers, and the promoters were defined with an arbitrarily selected 1000 bp extension downstream from the transcription start site, although the promoter lengths vary.

Finally, no other WGS data for DKD were available for replication of our findings, but we attempted replication in multiple datasets, including studies with imputed GWAS data for DKD in type 1 diabetes, and GWAS, WES and WGS datasets of phenotypes for kidney disease from the general population. Due to these limitations, we report replication at nominal significance (*p*<0.05). Despite the limited replication available, replication at 18q12.3 (*METTL4*) rs16943099, *THAP7* rs369250, and *NAT16* rs34985488 remained significant after correcting for the total number of tested SNPs (*n*=17). In addition, evidence from eQTL and differential gene expression in kidney tissue, as well as the *LTA* rs2229092 association with circulating TNF receptor levels, supports the relevance of the novel loci identified here, especially for the 18q12.3 (*METTL4*) enhancer region and the *NAT16* and *LTA* genes. However, these genes and variants represent risk/protective alleles rather than high-penetrance variants, and further validation is needed to confirm their role in DKD.

## Supplementary Information

Below is the link to the electronic supplementary material.

ESM (PDF 1440 KB)

## Data Availability

The FinnDiane WES and WGS datasets generated and/or analysed during the current study are not publicly available as the participants’ written consent does not allow data sharing. Collaborations to research individual-level data may be proposed by correspondence with the lead investigator.

## Abbreviations

CKD: Chronic kidney disease
DKD: Diabetic kidney disease
ESKD: End-stage kidney disease
eQTL: Expression quantitative trait locus
GWAS: Genome-wide association study
LT-α: Lymphotoxin-α
MAC: Minor allele count
MAF: Minor allele frequency
PAV: Protein-altering variant
PC: Principal component
PTV: Protein-truncating variant
TNFR: TNF receptor
WES: Whole-exome sequencing
WGS: Whole-genome sequencing

## Authors’ relationships and activities

P-HG has served on advisory boards for AbbVie, Astellas, AstraZeneca, Bayer, Boehringer Ingelheim, Eli Lilly, Janssen, Medscape, MSD, Mundipharma, Novartis, Novo Nordisk and Sanofi, and has received lecture honoraria from Astellas, AstraZeneca, Bayer, Boehringer Ingelheim, Eli Lilly, Elo Water, Medscape, MSD, Mundipharma, Novartis, Novo Nordisk and Sanofi. P-HG has also received investigator-initiated grants from Eli Lilly and Roche. The remaining authors declare that there are no relationships or activities that might bias, or be perceived to bias, their work.
