# Supplementary material for: Whole-exome and whole-genome sequencing of 1064 individuals with type 1 diabetes reveals novel genes for diabetic kidney disease
Source: Diabetologia. 2024 Aug 6;67(11):2494–506. doi: 10.1007/s00125-024-06241-1 (PMC11519100; doi:10.1007/s00125-024-06241-1)
Supplement: Supplementary file 1 — ESM (PDF 1440 KB) [file 125_2024_6241_MOESM1_ESM.pdf]

ELECTRONIC SUPPLEMENTARY MATERIAL (ESM)

Whole-exome and Whole-genome Sequencing of 1064 Individuals with Type 1 Diabetes Reveals Novel Genes for Diabetic Kidney Disease

Jani K Haukka[1,2,3], Anni A Antikainen[1,2,3], Erkka Valo[1,2,3], Anna Syreeni[1,2,3], Emma H Dahlström[1,2,3], Bridget M Lin[4], Nora Franceschini[4], Andrzej S Krolewski[5,6], Valma Harjutsalo[1,2,3], Per-Henrik Groop[1,2,3,7], Niina Sandholm[1,2,3] on behalf of the FinnDiane Study Group

[1] Folkhälsan Institute of Genetics, Folkhälsan Research Center, Helsinki
[2] Department of Nephrology, University of Helsinki and Helsinki University Hospital, Helsinki, Finland
[3] Research Program for Clinical and Molecular Metabolism, Faculty of Medicine, University of Helsinki, Helsinki, Finland
[4] Department of Epidemiology, University of North Carolina, Chapel Hill, NC, United States
[5] Section on Genetics and Epidemiology, Research Division, Joslin Diabetes Center; Boston, MA, 02215, USA
[6] Department of Medicine, Harvard Medical School; Boston, MA, 02215, USA.
[7] Department of Diabetes, Central Clinical School, Monash University, Melbourne, Victoria, Australia

ESM Figures: 5

ESM Tables: 14

Contents

ESM Fig. 1 - Genetic principal components (PC) 1 and 2 plotted for A) WGS and B) WES 3

ESM Fig. 2 – Power analysis. 4

ESM Fig. 3 – *METTL4* enhancer region rs1694309* is associated with DKD and alters Mafb binding affinitiy 5

ESM Fig. 4 - rs16943099 (*METTL4*) association with DKD stratified by sex. 6

ESM Fig. 5 – Locuszoom on chromosome 7:101.0-101.5Mb 7

ESM Table 1 – Variant function classes from SNPEff annotation, used for the gene aggregate test 8

ESM Table 2 – WGS single variant analysis for DKD using Firth test (*n*=583) 9

ESM Table 3 – Single variant analysis replication in FinnDiane (*n*=3724) and THL (*n*=2131) GWASes for DKD, TOPMed WGS for CKD (*n*=23,732) 10

ESM Table 4 – Variant replication across FinnGen kidney disease phenotypes in the Finnish FinnGen general population GWAS data. 11

ESM Table 5 – eQTL, gene expression and HI-C capture data obtained from databases. 12

ESM Table 6 – Individual associations of PAVs and PTVs inside suggestively DKD-associated genes 14

ESM Table 7 – Replication of suggestive gene aggregate results 15

ESM Table 8 – HLA haplotypes in FinnDiane WES/WGS cohorts and their associated HLA-based type 1 diabetes risk classes 16

ESM Table 9 - Clinical characteristics of the *LTA* rs2229092 minor allele carriers in individuals with OLINK proteomic data available within the combined WES and WGS data sets 17

ESM Table 10 – Lookup of monogenic kidney disease-causing genes on WES-WGS meta-analysis for DKD 18
ESM Table 11 –Tested variants in the associated sliding-window, promoter and enhancer associations (N=583) 21
ESM Table 12 – Single variant lead variant associations adjusted for clinical covariates 23
ESM Table 13 – The 17 lead variants from single variant, gene aggregate, sliding-window and regulome analysis tested for phenotype*sex interaction and more severe ESKD phenotype 24
ESM Table 14 - FinnDiane physicians and nurses participating in the collection of the FinnDiane study subjects 25
REFERENCES 28

ESM Fig. 1 - Genetic principal components (PC) 1 and 2 plotted for a) WGS and b) WES suggest that after the sample and variant QC, there were no significant outlier samples and no detectable ancestry clusters. The PC1 explained 0.69% and PC2 0.63% of variance within WES and WGS.

a

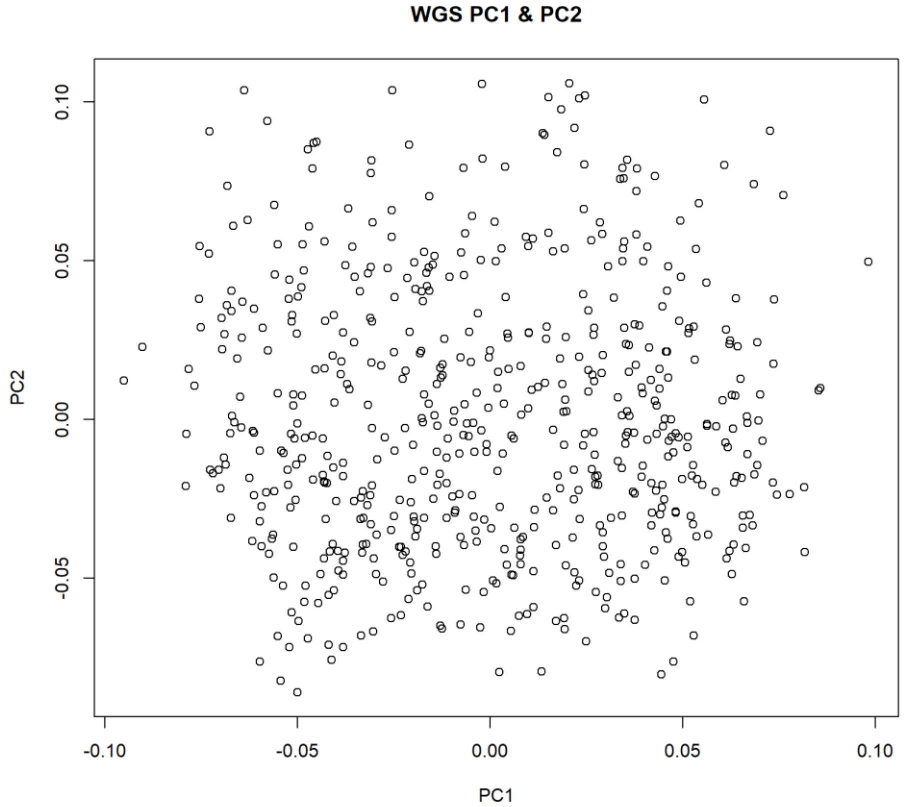

b

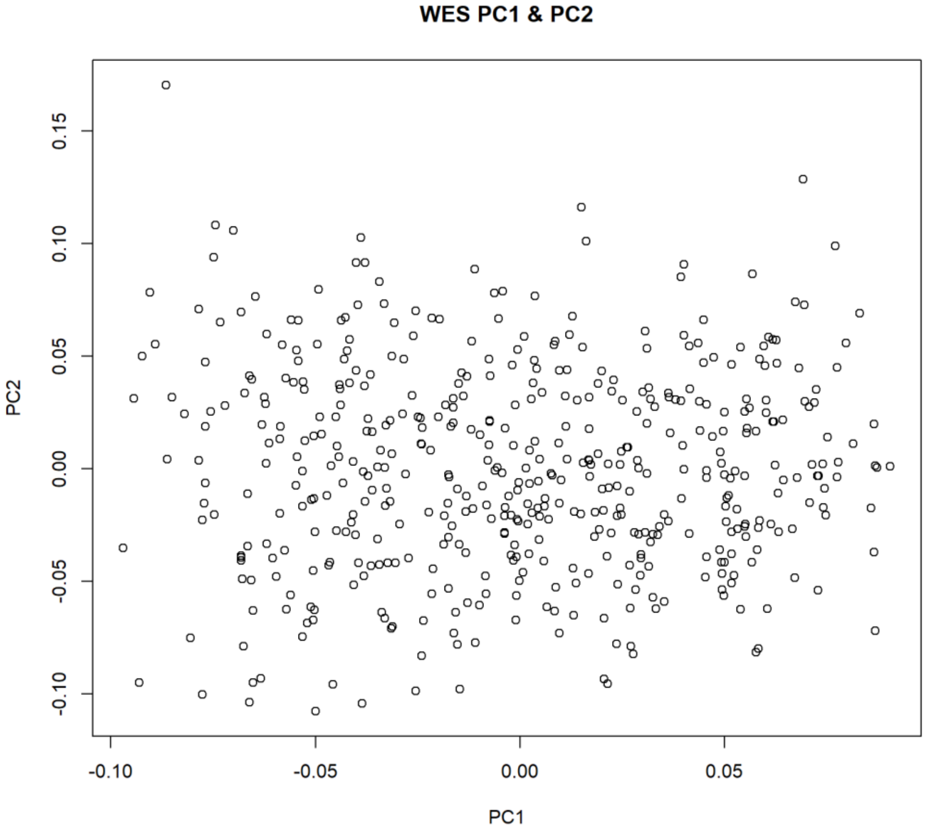

ESM Fig. 2 – Power analysis. Figures indicate the statistical power to detect an association with an odds ratio (OR) varying from 1.1 to 5.0, as a function of the minor allele frequency (MAF). Power calculations are based on the combined set of all WES + WGS samples (N=1064). **a**: power to detect an association with genome-wide statistical significance ($\alpha=5\times10^{-8}$). **b**: power to detect an association with suggestive significance ($\alpha=3.5\times10^{-5}$).

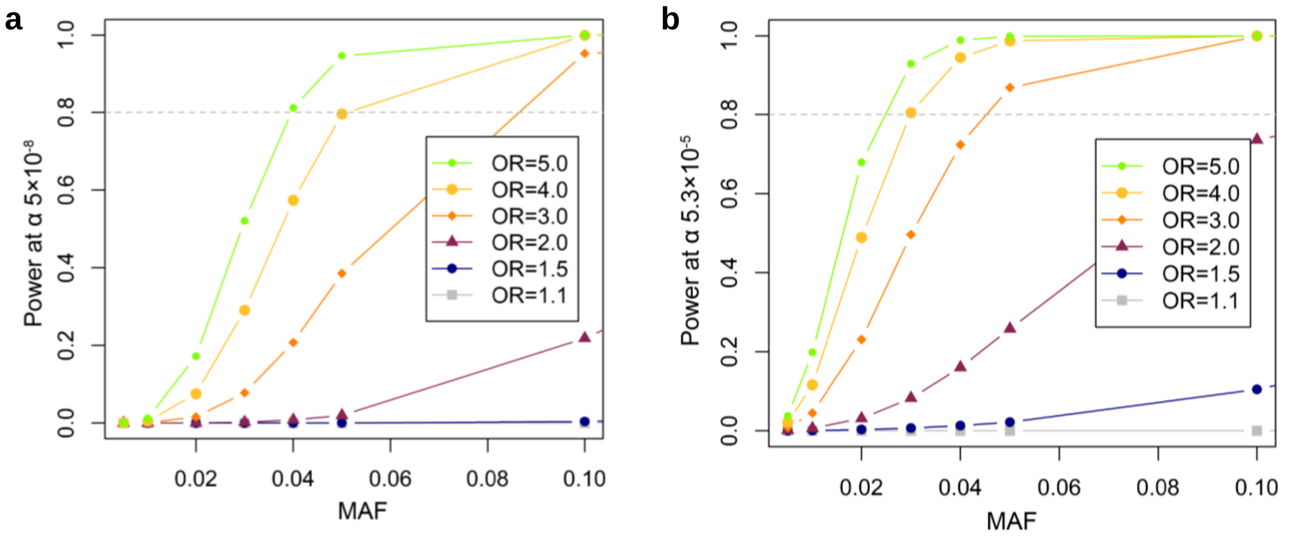

ESM Fig. 3 – *METTL4* enhancer region rs1694309* is associated with DKD and alters MafB binding affinitiy (**a**). Red color indicates TRAP predicted binding region for the JASPAR MA0117.1 motif. **b**: MafB and *METTL4* gene expression in human kidneys [1]. Figures plotted at http://humphreyslab.com/SingleCell/. **c** and **d**: in nephroseq database, Woroniecka et al. data for DKD vs healthy living donors, *MAFB* is underexpressed in DKD in glomeruli (**c**; p=4.6×10^{-4}, fold change -4.17) and overexpressed in tubuli (**d**; p=4.9×10^{-4}, fold change 2.25) [2].

a

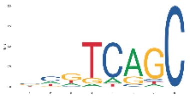


```
REF tattcattcaaaacaggattgagACGTCAGCttgacaaagctttctctcct
ALT tattcattcaaaacaggattgagaccctcagcttgacaaagctttctctcct
                           *
```

b

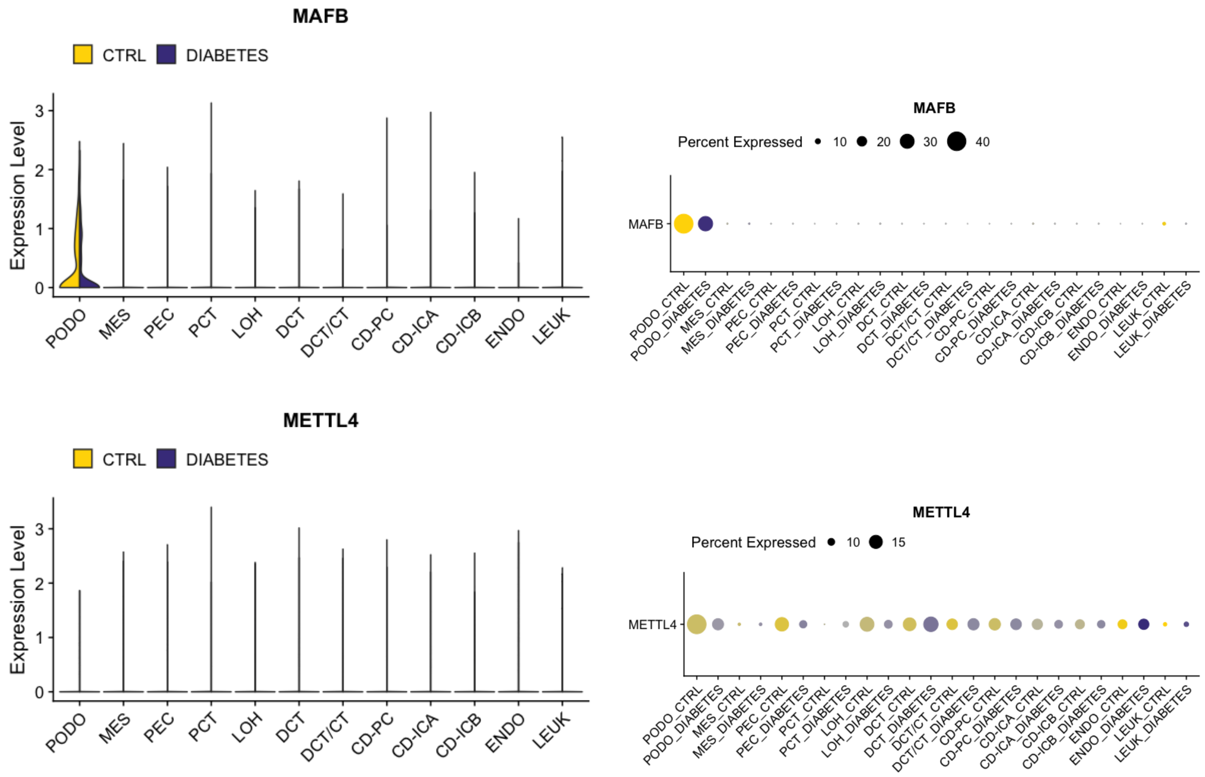

c

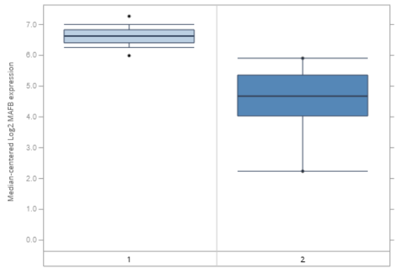

d

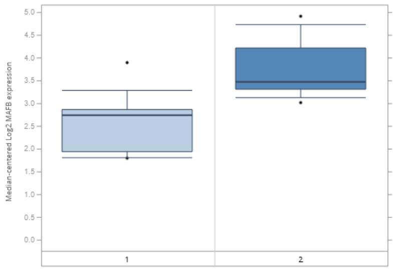

ESM Fig. 4 - rs16943099 (*METTL4*) association with DKD stratified by sex. rs16943099 was significantly associated with DKD in men (OR=0.55, 95% confidence interval [CI] 0.43 – 0.69) but not in women (OR=0.88, 95% CI 0.66 – 1.18).

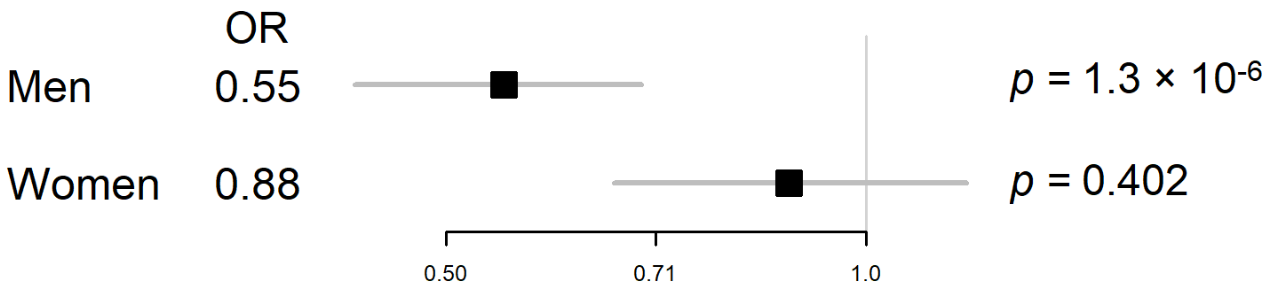

ESM Fig. 5 – Locuszoom on chromosome 7:101.0-101.5Mb. The highlighted *AP1S1* 3' UTR rs1048365 and *NAT16* p.Phe63Ser rs34985488 both have significant eQTL activity in tubule on *AP1S1*, *VGF* and *MOGAT3* (colored in orange).

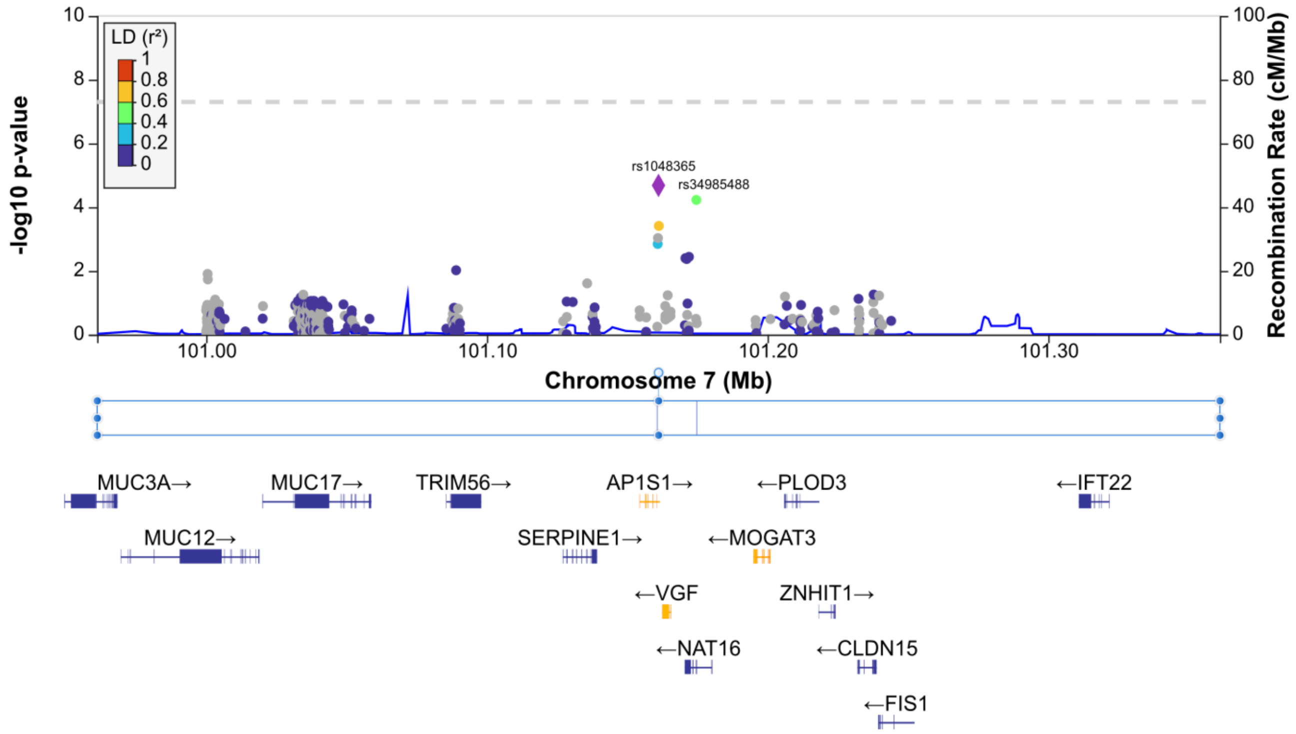

ESM Table 1 – Variant function classes from SNPEff annotation, used for the gene aggregate test. Protein-truncating variants (PTVs) include variants that are expected to severely or completely disrupt protein function, whereas protein-altering variants include the PTV, but also variants, such as missense variants and INDELs, that are expected to have less effect on protein function

Protein-altering variants (PAVs)	Protein-truncating variants (PTVs)
5_prime_UTR_premature_start_codon_gain_variant	start_lost
5_prime_UTR_truncation&exon_loss_variant	stop_gained
bidirectional_gene_fusion	stop_lost
gene_fusion	bidirectional_gene_fusion
conservative_inframe_deletion	gene_fusion
conservative_inframe_insertion	frameshift_variant
disruptive_inframe_deletion	exon_loss_variant
disruptive_inframe_insertion	splice_acceptor_variant
start_lost	splice_donor_variant
stop_gained	
stop_lost	
exon_loss_variant	
frameshift_variant	
missense_variant	
splice_acceptor_variant	
splice_donor_variant	
structural_interaction_variant	

ESM Table 2 – WGS single variant analysis for DKD using Firth test (*n*=583)

Position	rs#	Genes*	OR	AF	p value	Replication *p* value (FD GWAS)
14:92202604	rs66867671	30.5kb downstream from *CPSF2*	0.50	0.32	1.212×10^{-6}	0.846
14:92203776	rs12881014	31.6kb downstream from *CPSF2*	0.51	0.32	1.658×10^{-6}	0.814
14:92206163	rs61976635	34.0kb downstream from *CPSF2*	0.51	0.32	1.898×10^{-6}	0.814
14:92208383	rs35205959	36.2kb downstream from *CPSF2*	0.52	0.33	4.016×10^{-6}	0.880
14:92208614	rs61976640	36.5kb downstream from *CPSF2*	0.52	0.33	4.016×10^{-6}	0.887
14:92209896	rs11624199	37.8kb downstream from *CPSF2*	0.53	0.32	6.237×10^{-6}	0.901
16:36109142	.	Multiple RNA/ pseudogenes, closest to *AC116553.3*	2.84	0.10	4.362×10^{-6}	-
16:36109143	.	Multiple RNA/ pseudogenes, closest to *AC116553.3*	2.74	0.10	8.422×10^{-6}	-
19:17174120	rs181653048	*MYO9B* intron	0.39	0.11	9.395×10^{-6}	0.794
19:17174122	rs112921283	*MYO9B* intron	0.39	0.11	9.395×10^{-6}	0.794

OR: odds ratio for the minor allele. AF: Minor (=effect) allele frequency. P-value: P-value in WGS data using Firth test. Replication P-value (FD GWAS): P-value for association with DKD in the GWAS data for 3,724 non-overlapping FinnDiane participants with type 1 diabetes.

ESM Table 3 – Single variant analysis replication in FinnDiane (*n*=3724) and THL (*n*=2131) GWASes for DKD, TOPMed WGS for CKD (*n*=23,732)

Position	rsid	Gene[a]	CADD	SIFT	PolyPhen	Variant classification[B]	TOPMed MAF	TOPMed *p*	FinnDiane GWAS *p*	THLBB GWAS *p*
**SINGLE VARIANT META-ANALYSIS**										
2:61825245	rs3736598	*FAM161A*	11.7	-	-	-	0.38	0.19	0.43	0.68
2:61826155	rs6748320	*FAM161A*	1.8	-	-	-	0.38	0.19	0.43	0.68
22:21002277	rs369250	*THAP7*	5.27	-	-	-	0.44	0.71	0.17	0.61
7:101161149	rs1048365	*AP1S1*	2.75	-	-	-	0.25	0.92	0.76	NA
7:105107452	rs117986340	*KMT2E*	27.5	deleterious_lc	probably_damaging	Benign/ Uncertain Risk Allele	0.03	0.87	0.60	0.97
9:109176646	rs10979729	*EPB41L4B*	1.43	-	-	-	0.05	0.12	0.52	0.61
**GENE AGGREGATE META-ANALYSIS SNPS**										
17:75522195	rs200434678	*TSEN54*	24.9	deleterious	probably_damaging	VUS/ Uncertain Risk Allele	0.0003	0.62	0.89	NA
17:75523335	rs200228117	*TSEN54*	25.4	Tolerated	benign	VUS/ Uncertain Risk Allele	NA	NA	0.29	0.69
4:86849099	rs17694522	*SLC10A6*	22.5	deleterious	benign	VUS/ Uncertain Risk Allele	0.04	0.08	0.79	0.86
6:31572980	rs2229092	*LTA*	6.52	tolerated	benign	Benign/ Uncertain protective allele + functional support	0.04	0.52	0.27	0.35
7:101174620	rs34985488	*NAT16*	25.4	deleterious	possibly_damaging	VUS/ Uncertain Risk Allele	0.15	0.75	0.58	1.00
**SLIDING-WINDOW, PROMOTOR AND ENHANCER-WIDE ANALYSIS ON WGS DATA**										
18:2351193	rs16943099	*METTL4*	0.651	-	-	-	0.01	0.38	0.79	0.72
2:40755782	rs183413211	*LINC01794*	2.10	-	-	-	0.004	**0.04**	0.66	0.64
4:75124427	rs114761270	-	9.12	-	-	-	0.007	0.50	0.46	0.25
9:87792152	rs34407883	*CTSL*	0.837	-	-	-	0.001	0.98	NA	NA
9:87792755	rs12349827	*CTSL*	0.305	-	-	-	0.01	0.25	NA	0.94
9:87793063	rs12349926	*CTSL*	0.538	-	-	-	0.03	0.89	0.37	0.71

[A] Underlying, annotated or closest gene

[B] Variant classification for protein altering variants following ACMG/AMP classification as defined by InterVar (https://wintervar.wglab.org/results.php) [3]/ following the ClinGen Low Penetrance/Risk Allele Working Group [4].

ESM Table 4 – Variant replication across FinnGen kidney disease phenotypes in the Finnish FinnGen general population GWAS data.

**Position**	**rsid**	**Genes/ Region**	**CKD *p***	**Diabetic nephropathy *p***	**T1D with renal complications *p***	**T2D with renal complications *p***	**Other**
N cases			9073	4111	1579	2684	
N controls*			363,177	308,539	308,280	308,280	
**SINGLE VARIANT META-ANALYSIS**							
9:109176646	rs10979729	*EPB41L4B*	0.88	0.53	0.085	1.0	
2:61825245	rs3736598	*FAM161A*	0.46	0.26	0.96	0.33	
22:21002277	rs369250	*THAP7*	**$2.7×10^{-4}$**	**0.012**	0.27	**0.01**	
2:61826155	rs6748320	*FAM161A*	0.47	0.26	0.98	0.33	
7:101161149	rs1048365	*AP1S1*	NA	NA	NA	NA	
7:105107452	rs117986340	*KMT2E*	0.81	0.93	0.44	0.68	
**GENE AGGREGATE META-ANALYSIS SNPS**							
4:86849099	rs17694522	*SLC10A6*	0.97	0.27	0.71	0.35	
6:31572980	rs2229092	*LTA*	0.21	**0.027**	**0.0044**	0.88	T1D, wide definition p=$3.4×10^{-13}$, T1D with ophthalmic complications p=$4.5×10^{-13}$
7:101174620	rs34985488	*NAT16*	**0.0028**	0.16	0.32	0.48	
17:75522195	rs200434678	*TSEN54*	0.58	0.87	0.60	0.96	
17:75523335	rs200228117	*TSEN54*	0.65	0.75	0.97	0.71	
**SLIDING-WINDOW, PROMOTOR AND ENHANCER-WIDE ANALYSIS ON WGS DATA**							
9:87793063	rs12349926	*CTSL*	0.63	0.13	0.26	0.22	
9:87792152	rs34407883	*CTSL*	NA	NA	NA	NA	
9:87792755	rs12349827	*CTSL*	0.98	0.17	0.56	0.24	
2:40755782	rs183413211	*LINC01794*	0.27	0.72	0.96	0.30	
4:75124427	rs114761270	-	0.27	0.28	0.37	0.67	
18:2351193	rs16943099	*METTL4*	0.10	**0.036**	0.17	**$8.6×10^{-4}$**	

* Controls include also individuals without diabetes

ESM Table 5 – eQTL, gene expression and HI-C capture data obtained from databases. a Human kidney eQTL data for the top single variant analysis results, and for the top variants from the lead gene-aggregate test genes and genome-wide sliding window, promoter and enhancer analyses (for which variants with *P*<0.05 were tested) from the Human Kidney eQTL Atlas [5], **b** gene expression for the top meta-analysis genes on kidney other kidney conditions obtained from Nephroseq database (www.nephroseq.org/), **c** Hi-C capture the top enhancer regions (SNPs with p<0.05 within regions were queried) **d** Predicted allele-specific MafB binding activity of rs1694309, when the ±25bp sequence was queried with Transcription factor Affinity Prediction (TRAP) tool and jasper vertebrates and transfac_2010.1 vertebrates (human motifs only) databases.

a

SNP	Position	Gene	Direction	$\text{Effect}_{\text{ALT}}$	SE	*p*
rs1048365	7:100804430:C_T	*AP1S1*	++++	0.9991	0.0354	4.98×10^{-175}
rs1048365	7:100804430:C_T	*VGF*	++++	0.6245	0.0552	1.147×10^{-29}
rs1048365	7:100804430:C_T	*MOGAT3*	++-+	0.2295	0.0473	1.235×10^{-6}
rs34985488	7:100817901:A_G	*AP1S1*	+?++	0.9021	0.0584	8.056×10^{-54}
rs34985488	7:100817901:A_G	*VGF*	+?++	0.441	0.0741	2.646×10^{-9}
rs34985488	7:100817901:A_G	*MOGAT3*	+?-+	0.3036	0.058	1.644×10^{-7}
rs369250	22:21356566:A_G	*THAP7-AS1*	-?--	-0.6864	0.0489	8.488×10^{-45}

b

Phenotype	Gene	n (cases/controls)	P-value	t-test	Fold-change	Notes
MEMBRANOUS GLOMERULONEPHROPATHY	*LTA*	9/3	0.002	5.589	-1.641	
HODGIN DIABETES MOUSE GLOM	*SLC10A6*	7/5	0.002	3.894	2.334	
NAKAGAWA CKD KIDNEY	*TSEN54*	48/5	1.90×10^{-14}	-10.582	-5.181	Controls have increased expression of TSEN54, about half of cases have decreased, half increased expression

c

SNP	Tissue	Interacting regions (hg19)
rs16943099	Bladder	18:2349726-2356087 (enhancer): 18:2567644-2580208 (*METTL4*)
rs34407883	Bladder	9:90395315-90407069 (enhancer) : 9:90335670-90343626 (*CTSL*)

d

Database for Transcription factor binding site motifs	p_{REF}	p_{ALT}	Rank	Difference log(*p*) for two sequences	Matrix ID	Matrix name
**rs16943099 (G/C) (18q12.3)**						
TRANSFAC vertebrates	$<1.75\times10^{-6}$	0.239	1	5.13	V$MAFB_01	MafB
jaspar vertebrates	6.3×10^{-4}	0.255	1	2.61	MA0117.1	MafB
**rs12349926 (G/A) (9q21.11)**						
TRANSFAC vertebrates	0.004	$<4\times10^{-6}$	1	-3.03	M01665	Irf8_Q6
jaspar vertebrates	0.61	0.16	1	-0.59	MA0028.1	ELK1

p_{REF}/ p_{ALT}: P-value for the sequence forming a transcription factor binding motif, when the SNP has the REF/ALT allele. Rank: rank for the difference in the p-values for the two alternative SNP sequences. Matrix ID: transcription factor binding motif identifier. Matrix name: transcription factor binding matrix name (=transcription factor name).

ESM Table 6 – Individual associations of PAVs and PTVs inside suggestively DKD-associated genes

Gene (variant filters)	rsid	Position	A1	A2	MAF/MAC WGS*	MAF/MAC WES*	HWE *p* (WGS/WES)	OR [95% CI]	DKD *p*	Direction WES/WGS
***NAT16* (PAV 10%)**		7:101172173	a	g	MAC≤3		1/-	19.39 [0.3, 1232.69]	0.162	?+
	rs781324827	7:101172525	a	c	MAC≤3		1/-	0.24 [0, 17.09]	0.509	?-
	rs768356640	7:101172554	g	ggcagcggcgagaa ggtgccagag gtcc	MAC≤3		0.002/-	0.06 [0, 3.76]	0.185	?-
		7:101174511	a	c		MAC≤3	-/1	7.46 [0.14, 390.15]	0.320	+?
	rs34985488	7:101174620	a	g	0.09862	0.07449	0.82/0.33	1.89 [1.38, 2.57]	**5.85×10^{-5}**	++
		7:101174795	a	c		MAC≤3	-/1	19.39 [0.3, 1232.69]	0.434	+?
***SLC10A6* (PAV 10%)**	rs139158966	4:86823730	c	g	MAC≤3	MAC≤3	1/1	0.2 [0.01,3.43]	0.265	--
	rs770266438	4:86831834	a	c	MAC≤3		1/-	5.12 [0.29, 90.08]	0.475	?+
	rs17694522	4:86849099	a	g	0.06861	0.07347	0.75/0.32	0.52 [0.37,0.73]	**1.78×10^{-4}**	++
***TSEN54* (PAV 1%)**		17:75517212	a	c	MAC≤3			0.52 [0.37, 0.73]	0.462	?-
	rs758995350	17:75517582	a	c		MAC≤3	-/1	4.7 [0.08, 290.59]	0.249	+?
	rs770897552	17:75519014	c	g	MAC≤3		1/-	5.36 [0.31, 93.03]	0.523	?+
	rs200015685	17:75521847	a	g	MAC≤3		1/-	0.24 [0, 18.83]	0.175	?-
	rs762725861	17:75521901	a	g	MAC≤3		1/-	0.06 [0, 3.54]	0.463	?-
	rs777546135	17:75521908	t	g		MAC≤3	-/1	4.7 [0.08, 292.22]	0.377	+?
		17:75521978	t	g		MAC≤3	-/1	5.99 [0.11, 318.78]	0.308	+?
		17:75522000	t	g			-/1	7.77 [0.15, 398.75]	0.231	+?
		17:75522059	t	c	MAC≤3		1/	11.58 [0.21, 634.97]	0.136	?+
		17:75522194	c	g		MAC≤3	-/1	25.37 [0.36, 1774.56]	0.231	+?
	rs200434678	17:75522195	a	g	MAC≤3		1/-	11.57 [0.21, 632.84]	**0.042**	?+
		17:75522294	a	g	MAC≤3		1/	21.75 [1.12, 422.07]	0.452	?+
	rs200228117	17:75523335	a	g	MAC≤3	MAC≤3	1/1	4.88 [0.08, 303.87]	**0.025**	++
		17:75524351	a	g		MAC≤3	-/1	10.42 [1.34, 80.85]	0.214	+?
***LTA* (PAV 5%)**	rs2229092	6:31572980	a	c	0.03434	0.03556	0.50/1	0.39 [0.24, 0.63]	**1.48×10^{-4}**	--

A1/A2: minor/major alleles. HWE P (WGS/WES): P-value for Hardy Weinberg equilibrium in WGS/ WES data. OR [95% CI]: odds ratio and 95% confidence interval for the minor (A2) allele. P-value: p-value for association with DKD. Direction WES/WGS: direction of effect (for the minor allele) in the WES/WGS data set, ? denotes that the variant was not available in the data set.

* Minor allele count (MAC) is reported instead of minor allele frequency (MAF) for extremely rare variants (MAC≤3) to protect rare variant carriers' privacy and enhance the table readability

ESM Table 7 – Replication of suggestive gene aggregate results

GENE	MAF-class	MetaSKAT *p*	T2D UKBB_Nephropathy_loose p	TOPMED *p*	FD GWAS *p*	THLBB *p*
NAT16	0.1	1.4×10^{-4}	0.316296	0.5555	0.5813022	NA
LTA	0.05	1.5×10^{-4}	NA	NA	NA	0.4295364
SLC10A6	0.1	2.7×10^{-4}	0.162221	0.5622	0.8385422	0.7177943
TSEN54	0.01	3.7×10^{-4}	0.438022	0.7239	0.8519525	0.7194490

ESM Table 8 – HLA haplotypes in FinnDiane WES/WGS cohorts and their associated HLA-based type 1 diabetes risk classes

Haplotype	Type 1 diabetes OR [6]	N FinnDiane WES/WGS	HLA type 1 diabetes risk
DRB1*0405-DQA1*0301G-DQB1*0302	11.37	≤3	High
DRB1*0401-DQA1*0301G-DQB1*0302 (DR4)	8.39	684	High
DRB1*0301-DQA1*0501G-DQB1*0201 (DR3)	3.64	459	High
DRB1*0404-DQA1*0301G-DQB1*0302	1.59	194	Neutral
DRB1*0801-DQA1*0401G-DQB1*0402	1.25	163	Neutral
DRB1*1302-DQA1*0102G-DQB1*0604	0.87	92	Neutral
DRB1*0101-DQA1*0101G-DQB1*0501	0.71	209	Low
DRB1*0102-DQA1*0101G-DQB1*0501	0.66	≤3	Low
DRB1*0901-DQA1*0301G-DQB1*0303	0.53	63	Low
ALL REMAINING HAPLOTYPES IN FINNDIANE WES/WGS	OR<0.50	176	Low

TYPE 1 DIABETES OR [6]: haplotype OR for type 1 diabetes as reported in Erlich et al. 2008.

N FINNDIANE WES/WGS: number of FinnDiane WES/WGS participants carrying the haplotype.

ESM Table 9 - Clinical characteristics of the *LTA* rs2229092 minor allele carriers in individuals with OLINK proteomic data available within the combined WES and WGS data sets

	AA carriers	C/A, C/C carriers	*p* value
n	120	8	
DKD class (n)			
Microalbuminuria	11	2	
Severe albuminuria	109	6	
ESKD	0	0	
Sex (% Women)	40.3	37.5	NS
Age at type 1 diabetes onset (years)	11.1 [7.1, 14.8]	10.5 [5.9, 14.8]	NS
Duration of diabetes (years)	23.8 [19.2, 30.2]	22.3 [17.6, 27.6]	
HbA1c (%)	9.00 [7.97, 10.10]	8.95 [7.70, 9.62]	NS
HbA1c (mmol/mol)	75 [64, 87]	73 [61, 82]	NS
BMI (kg/m^2)	25.2 [22.3, 27.7]	25.7 [22.2, 29.1]	NS
SBP	139 [130, 152]	134 [130, 153]	NS
DBP	82 [79, 91]	91 [81, 93]	NS
Total cholesterol (mmol/L)	5.32 [4.78, 5.93]	5.56 [4.75, 6.04]	NS
Triglycerides (mmol/L)	1.22 [0.92, 1.71]	1.42 [0.96, 1.75]	NS
Smokers (%)	56.1	75.0	NS
TNF-R1 (NPX)*	9.24 [8.80, 9.74]	8.64 [8.49, 8.70]	0.009
TNF-R2 (NPX)*	9.25 [9.04, 9.97]	8.89 [8.59, 8.99]	0.006
TNF-R3 (NPX)*	11.55 [11.32, 12.12]	11.01 [10.89, 11.26]	0.024

Values are given as median and [inter-quartile range]. Characteristics are given for the time-point closest to the date based on available longitudinal data (HbA1c, BMI, SBP and DBP). Smoking status (current or former smoker vs. never smoker) and lipid values are recorded at the study baseline.

* The TNF-receptor levels are shown as relative Normalized Protein eXpression (NPX) values, and relative levels of different proteins aren’t comparable.

ESM Table 10 – Lookup of monogenic kidney disease-causing genes on WES-WGS meta-analysis for DKD. a meta-analysis p-values for all nominally (*P*<0.05) DKD-associated genes at MAF<0.1, MAF<0.05 and MAF<0.01, and **b** nominally significant genes highlighted among all the tested genes in different kidney disease categories.

a

**Gene**	**Coordinates**	***p*(MAF=0.1)**	***p*(MAF=0.05)**	***p*(MAF=0.01)**
**CYSTIC KIDNEY DISEASE OR NEPHRONOPHTHISIS**				
CCDC28B	1:32200595-32205453	0.779	0.779	**0.009**
CEP104	1:3812086-3857396	0.221	0.196	**0.007**
CEP164	11:117314557-117413266	0.113	0.114	**0.022**
CSPP1	8:67062417-67196778	0.093	**0.030**	0.238
DCDC2	6:24171755-24358059	**0.018**	**0.018**	0.119
DDX59	1:200623896-200669907	0.686	0.686	0.680
HOXA4	7:27128507-27130780	**0.0008**	**0.0008**	0.545
IFT122	3:129429607-129520510	**0.045**	**0.044**	0.075
IQCB1	3:121769761-121835079	**0.015**	**0.009**	0.386
KIF14	1:200551497-200620751	**0.002**	**0.002**	**0.026**
UMOD	16:20333051-20356301	0.366	0.366	**0.029**
**SYNDROMIC CAKUT**				
ATXN10	22:45671798-45845307	**0.026**	**0.026**	**0.026**
BSCL2	11:62689289-62709845	**0.023**	**0.023**	**0.023**
EP300	22:41092592-41180077	**0.048**	0.078	0.332
ERCC8	5:60866454-60945073	**0.026**	**0.026**	**0.026**
FANCA	16:89726683-89816977	0.376	0.244	**0.022**
FAT4	4:125314918-125492932	**0.041**	**0.032**	0.384
HNRNPUL2-BSCL2	7:150944961-150978321	**0.027**	**0.027**	**0.027**
KCNH2	2:113215997-113278921	0.311	0.236	**0.034**
PAX8	3:52075226-52154690	**0.021**	**0.021**	1.000
POC1A	20:51782331-51802521	**0.002**	**0.002**	**0.002**
SALL4	4:6269849-6303265	**0.035**	**0.035**	0.524
WFS1	22:45671798-45845307	0.207	0.210	**0.039**
	**ISOLATED CAKUT**			
DSTYK	1:205142505-205211702	**0.024**	**0.038**	**0.026**
SRGAP1	12:63843761-64162217	0.428	0.428	**0.012**
**CHRONIC LOMERULONEPHRITIS**				
**RENAL TUBULOPATHIES**				
SCN4A	17:63938554-63972918	0.413	0.413	**0.037**
SCNN1A	12:6346843-6377730	**0.015**	**0.015**	0.893
SCNN1G	16:23182745-23216883	**0.042**	**0.042**	**0.042**
WNK4	17:42780610-42797066	**0.034**	0.088	0.118
**NEPHROLITHIASIS OR NEPHROCALCINOSIS**				
CLDN16	3:190322541-190412138	**0.015**	**0.015**	0.150

b

**Cystic kidney disease or nephronophthisis**				NPHP1	*INVS\NPHP2*	*NPHP3*	
	NPHP4	***IQCB1\NPHP5***		*CEP290\NPHP6*		*GLIS2\NPHP7*	
	RPGRIP1L\NPHP8		*NEK8\NPHP9*	*SDCCAG8\NPHP10*		*TMEM67\NPHP11*	
	TTC21B\NPHP12		*WDR19\NPHP13*		*ZNF423\NPHP14*		
	CEP164\NPHP15		*ANKS6\NPHP16*		*IFT172\NPHP17*		
	CCDC41\CEP83		***DCDC2\NPHP19***		*MAPKBP1\NPHP20*		
	IFT81\CDV-1	*TRAF3IP1\SLS9*		*XPNPEP3\NPHPL1*		*FAN1\MTMR15*	
	PKHD1\ARPKD		*INPP5E\JBTS1*		*TMEM216\JBTS2*		
	AHI1\JBTS3	*ARL13B\JBTS8*		*CC2D2A\JBTS9*		*KIF7\JBTS12*	
	TCTN1\JBTS13		*TMEM237\JBTS14*		*CEP41\TSGA14*		
	TMEM138\JBTS16		*C5orf42\JBTS17*		*TCTN3\JBTS18*		
	TMEM231\JBTS20		***CSPP1\JBTS21***		*PDE6D\JBTS22*		
	KIAA0586\JBTS23		*TCTN2\JBTS24*		***CEP104\JBTS25***		
	KIAA0556\JBTS26		*MKS1\MKS1*	*B9D1\MKS9*	*B9D2\MKS10*	***KIF14\MKS12***	
	TMEM107\MKS13		*BBS1\BBS1*	*BBS2\BBS2*	*ARL6\BBS3*	*BBS4\BBS4*	
	BBS5\BBS5	*MKKS\BBS6*	*BBS7\BBS7*	*TTC8\BBS8*	*PTHB1\BBS9*	*BBS10\BBS10*	
	TRIM32\BBS11		*BBS12\BBS12*	*WDPCP\BBS15*		*LZTFL1\BBS17*	
	BBIP1\BBS18	*IFT27\BBS20*	*DDX59\OFD5*	*SCLT1\OFD9*	*C2CD3\OFD14*		
	KIAA0753\OFD15		***IFT122\CED1***		*WDR35\CED2*		
	IFT43\CED3	*IFT80\SRTD2*	*DYNC2H1\SRTD3*		*NEK1\SRTD6*	*WDR60\SRTD8*	
	IFT140\SRTD9		*WDR34\SRTD11*		*CEP120\SRTD13*		
	IFT57	*IFT52\SRTD*	*ALMS1\ALMS*	*PIK3R4\VPS15*		*TXNDC15*	
	SLC41A1	*POC1B*	*HSD17B4\MFP2*		*USH2A*	***UMOD***	
	HOXA4	*HOXB6*	*TBC1D32*	**CCDC28B**	EVC	EVC2	
	OFD1						
**Syndromic CAKUT**		*B3GALTL*	*BBS6*	*BBS8*	*BSCL2*	*CD151*	
	CD96	*CEP290*	*CHRNG*	*CISD2*	*CTU2*	*CYP21*	
	DACH1	*DHCR7*	*EMG1*	***ERCC8***	*ESCO2*	*ETFA*	
	ETFB	*ETFDH*	*FANCA*	*FANCB*	*FANCD2*	*FANCE*	
	FANCI	*FANCL*	***FAT4***	*FOXP1*	*HES7*	*HYLS1*	*ICK*
	IFT46	*IFT74*	*INPP5E*	*ITGA3*	*JAM3*	*LFNG*	
	LMNA	*LRIG2*	*LRP2*	*LRP4*	*MESP2*	*MKS3*	
	PEX5	*PMM2*	***POC1A***	*PROK2*	*RECQL4*	*ROR2*	
	RPS19	*SCARF2*	*STRA6*	*TMCO1*	*TWIST2*	*UBR1*	
	PEX1	*PIGL*	*PIGO*	*PIGN*	*PIGT*	*PIGV*	*PIGY*
	PTF1A	***WFS1***	*WNT3*	*ZMPSTE24*	*ACTB*	*ACTG1*	
	AIFM3	***ATXN10***	*BICC1*	*BMP7*	*BRAF*	*CDC5L*	
	CREBBP	*DACT1*	***EP300***	*ESRRG*	*FBN1*	*FGFR1*	
	FGFR3	*FGF10*	*FGF8*	*FGF3*	*FMN1*	*FOXC1*	
	FOXF1	*GDF3*	*GDNF*	*GFRA1*	*GLI2*	*HOXA13*	
	HOXD13	*JAG1*	*KAT6B*	*KCTD1*	***KCNH2***	*KRAS*	
	LMX1B	*LPP*	*MAP2K1*	*MAP2K2*	*MLL2/ KMT2D*		
	MYCN	*NFIX*	*NOTCH2*	***PAX8***	*PKD1*	*PKD2*	
	PROKR2	*PTPN11*	*RAF1*	*RAI1*	***SALL4***	*SEMA3A*	
	SEMA3E	*SETBP1*	*SHH*	*SF3B4*	*SNAP29*	*SOS1*	
	SOX9	*SRCAP*	*TBX1*	*TBX3*	*TFAP2A*	*TP63*	
	TRPS1	*TSC1*	*TSC2*	*WNT5A*	*ARID1B*	*DIS3L2*	
	FGFR2	*GDF6*	*GLI3*	*PCSK5*	*PTEN*	*RPS24*	
	VANGL1	*AXIN1*	*H19*	*KCNQ1OT1*	*NIPBL*	*CDKN1C*	
	CHD7	*AMER1*	*ATP7A*	*BCOR*	*DLG3*	*FAM58A*	

	FLNA	*GPC3*	*MID1*	*NSDHL*	*OFD1*	*PIGA*	
	PORCN	*SMC1A*	*UPF3B*	*ZIC3*	*GDF11*	*OSR1*	
	TTC30A	*UBE3A*	*SH2B1*				
**Isolated CAKUT**		*ACE*	*AGT*	*AGTR1*	*CHRM3*	*ETV4*	
	FRAS1	*FREM1*	*FREM2*	*GRIP1*	*HPSE2*	*ITGA8*	*REN*
	TRAP1	*FGF20*	*BMP4*	*CHD1L*	*CRKL*	***DSTYK***	*EYA1*
	GATA3	*GREB1L*	*HNF1B*	*MUC1*	*NRIP1*	*PAX2*	
	PBX1	*RET*	*ROBO2*	*SALL1*	*SIX1*	*SIX2*	*SIX5*
	SLIT2	*SOX17*	***SRGAP1***	*TBX18*	*TNXB*	*UPK3A*	
	WNT4	*KAL1*					
**Chronic glomerulonephritis**			*ADAMTS13*	*CFI*	*COL4A4*	*CFB*	
	CFHR3	*CFHR5*	*EIF2AK3*	*FN1*	*FOXC2*	*GSN*	*LYZ*
	THBD	*SPRY2*	*C3*	*CD46*	*CFH*	*CFHR1*	
	COL4A3	*COL4A5*	*COL4A6*				
**Renal tubulopathies**		*ATP6B1*	*ATP6V1C2*	*BCS1L*	*BSND*	*COG6*	
	COQ9	*CYP11B1*	*CYP17A1*	*CYP27B1*	*EGF*	*FGF23*	
	GALNT3	*HSD11B2*	*MRPS22*	*RRM2B*	***SCNN1A***	*SCNN1B*	
	SLC12A3	*SLC26A4*	*SLC2A2*	*SLC4A4*	*SLC4A5*	*SLC6A19*	
	SLC7A7	*SUCLA2*	*TRPM6*	*VIPAR*	*VIPAS39*	*WNK1*	
	WNK4	*AP2S1*	*AVP*	*CACNA1S*	*CNNM2*	*CUL3*	
	EHHADH	*FXYD6-FXYD2*		*GNA11*	*KCNJ5*	*KLHL3*	
	NR3C2	*SAC (ADCY10)*		***SCN4A***	*CLCNKA*	*CLCKNB*	
	SLC36A2	*SLC6A20*	*AQP2*	***SCNN1G***	*SLC5A2*	*AVPR2*	
**Nephrolithiasis or nephrocalcinosis**			*AGXT*	*ALDOB*	*ALPL*	*APRT*	
	ATP6V0A4	*ATP6V1B1*	*ATP7B*	*CA2*	*CLCNKB*	***CLDN16***	
	CLDN19	*CTNS*	*CYP24A1*	*FAH*	*FAM20A*	*G6PC*	
	GRHPR	*HOGA1*	*KCNJ1*	*KCNJ10*	*SLC12A1*	*SLC26A1*	
	SLC2A2	*SLC34A3*	*XDH*	*HNF4A*	*SLC9A3R1*	*CASR*	
	SLC22A12	*SLC2A9*	*SLC34A1*	*SLC3A1*	*SLC4A1*	*SLC7A9*	*VDR*
	CLCN5	*HPRT1*	*OCRL*				
**Nephrotic syndrome**		*ADCK4*	*ALG1*	*APOA1*	*ARHGDIA*	*AVIL*	
	CD2AP	*COQ2*	*COQ6*	*CUBN*	*CRB2*	*DGKE*	
	EMP2	*FAT1*	*ITGA3*	*ITGB4*	*KANK1*	*KANK2*	
	KANK4	*LAGE3*	*LAMB2*	*LCAT*	*MAGI2*	*MYO1E*	
	NEU1	*NPHS1*	*NPHS2*	*NUP107*	*NUP133*	*NUP205*	
	NUP85	*NUP93*	*OSGEP*	*PDSS2*	*PLCE1*	*PTPRO*	
	SCARB2	*SGPL1*	*SMARCAL1*	*TP53RK*	*TPRKB*	*TTR*	
	VPS33B	*WDR73*	*XPO5*	*ACTN4*	*ANLN*	*ARHGAP24*	*INF2*
	LMX1B	*MYH9*	*PODXL*	*TRPC6*	*WT1*	*IKBKAP*	
	NXF5	*APOE*	*APOL1*	*GPC5*	*SYNPO*		

ESM Table 11 –Tested variants in the associated sliding-window, promoter and enhancer associations (*n*=583)

rEGULATORY REGION	rsid	Position	A1	A2	HWE P	MAC*	OR [95% CI]*	Firth p
**SLIDING-WINDOW 12Q14.3**	rs575374987	12:108104058	C	T	1	≤3	0.24 [0, 18.14]	0.829
		12:108104432	C	A	1	≤3	0.08 [0, 4.54]	0.416
	rs561150045	12:108104469	G	A	1	≤3	0.23 [0, 16.65]	0.808
	rs190528531	12:108105375	G	A	1	≤3	0.06 [0, 3.75]	0.376
	rs74676695	12:108106003	G	A	1	≤3	0.06 [0, 3.78]	0.377
	rs78452498	12:108106081	C	A	1	4	0.11 [0.01, 0.91]	0.147
		12:108106137	TTAATAA	T	1	≤3	0.11 [0.01, 2.22]	0.378
	rs531800089	12:108106452	C	A	1	≤3	0.06 [0, 3.7]	0.377
		12:108106913	C	T	1	≤3	0.24 [0, 16.9]	0.811
		12:108106970	C	T	1	≤3	0.05 [0, 1.02]	0.237
		12:108107279	G	C	1	≤3	0.05 [0, 3.23]	0.348
	rs192506403	12:108107522	C	T	1	≤3	0.23 [0.02, 2.68]	0.494
		12:108107725	T	C	1	≤3	0.05 [0, 3.02]	0.334
	rs117656942	12:108107793	C	T	1	≤3	0.12 [0.01, 2.25]	0.383
**SLIDING-WINDOW 4Q22.3**	rs151208036	4:75122307	G	A	1	≤3	5.24 [0.09, 298.6]	0.678
	rs376478683	4:75122408	AGAAG	A	1	≤3	4.41 [0.06, 302.08]	0.785
		4:75122522	GA	G	1	≤3	0.05 [0, 3.26]	0.348
		4:75122526	A	G	1	≤3	0.06 [0, 3.82]	0.380
		4:75122863	G	A	1	4	11.04 [1.38, 88.52]	0.113
		4:75123692	GCTTT	G	1	≤3	4.63 [0.24, 88.1]	0.610
		4:75123736	CA	C	1	≤3	4.54 [0.07, 287.16]	0.756
		4:75123739	A	C	1	≤3	4.55 [0.07, 287.83]	0.756
		4:75123742	G	T	1	≤3	0.38 [0.03, 4.1]	0.513
	rs72662456	4:75124048	C	T	1	≤3	23.85 [0.35, 1634.36]	0.326
		4:75124093	C	T	1	≤3	2.15 [0.11, 41.42]	0.626
		4:75124193	A	G	1	≤3	4.93 [0.45, 53.63]	0.419
	rs114761270	4:75124427	C	T	1	7	8.01 [1.64, 39.14]	**0.040**
	rs528483191	4:75124599	CTA	C	1	4	0.74 [0.09, 5.9]	0.775
	rs140007606	4:75124646	C	T	1	6	8.08 [1.44, 45.39]	0.058

	rs747665362	4:75124654	C	G	1	≤3	33.63 [0.43, 2615.88]	0.293
	rs770601133	4:75124950	T	G	1	10	0.66 [0.17, 2.54]	0.583
		4:75125317	A	C	1	≤3	9.62 [0.52, 176.62]	0.344
		4:75125568	C	T	1	≤3	0.23 [0, 16.65]	0.808
	rs147887794	4:75125585	A	C	1	≤3	4.66 [0.24, 88.99]	0.607
	rs62314936	4:75125980	T	A	1	7	4.86 [1.01, 23.27]	0.101
**PROMOTER 9Q21.11 (*CTSL3P*)**		9:87792152	C	CAG	1	40	0.34 [0.17, 0.67]	**0.003**
	rs141341525	9:87792611	G	A	1	5	1.47 [0.23, 9.33]	0.738
	rs12349827	9:87792755	G	T	1	8	0.13 [0.03, 0.58]	**0.030**
		9:87793023	G	C	1	≤3	0.23 [0, 16.04]	0.794
	rs12349926	9:87793063	G	A	1	23	0.17 [0.07, 0.42]	$\mathbf{6.08\times10^{-4}}$
	rs566499267	9:87793119	A	G	1	≤3	9.26 [0.5, 170.91]	0.356
**PROMOTER 2Q14.2 (*LINC01794*)**	rs191082134	2:40755328	C	T	1	≤3	0.07 [0, 4.25]	0.403
		2:40755562	A	T	1	≤3	0.23 [0, 16.73]	0.808
		2:40755569	G	C	1	≤3	0.05 [0, 3.11]	0.340
	rs183413211	2:40755782	T	A	1	6	0.08 [0.01, 0.46]	**0.036**
		2:40756209	TA	T	1	4	0.09 [0.01, 0.58]	0.068
**ENHANCER 18Q12.3 (*METTL4*)**	rs530496044	18:2351117	A	G	1	≤3	19.54 [0.31, 1246.66]	0.349
	rs16943099	18:2351193	G	C	1	15	0.16 [0.07, 0.38]	$\mathbf{2.09\times10^{-4}}$
**ENHANCER 9Q21.11 (*CTSL*)**		9:87793023	G	C	1	≤3	0.23 [0, 16.04]	0.794
	rs12349926	9:87793063	G	A	1	23	0.17 [0.07, 0.42]	$\mathbf{6.08\times10^{-4}}$
	rs566499267	9:87793119	A	G	1	≤3	9.26 [0.5, 170.91]	0.356
	rs549934850	9:87793172	G	A	1	≤3	0.23 [0, 15.48]	0.787

A1/A2: Major/minor allele . HWE P: P-value for Hardy Weinberg equilibrium. OR [95% CI]: odds ratio and 95% confidence interval for the minor (A2) allele. Firth P-value: Firth test p-value for association with DKD.

* Minor allele count (MAC) is reported as MAC≤3 for extremely rare variants to protect rare variant carriers' privacy

ESM Table 12 – Single variant lead variant associations adjusted for clinical covariates (OR [95% CI], *p*-value).

Position	rsid	Gene*	DKD OR	*p* DKD	HbA_{1c}	DBP	SBP	Cholesterol	triacylglycerol	BMI	Smoking status
**Single variant analysis**											
2:61825245	rs3736598	*FAM161A*	0.67 [0.56,0.81]	1.30×10^{-5}	0.7 [0.58,0.85], p=4.04×10^{-4}	0.66 [0.54,0.8], p=4.12×10^{-5}	.68 [0.56,0.82], p=5.60×10^{-5}	0.69 [0.57,0.84], p=2.94×10^{-4}	0.72 [0.58,0.89], p=0.003	0.69 [0.57,0.84], p=1.39×10^{-4}	0.69 [0.57,0.84], p=2.45×10^{-4}
2:61826155	rs6748320	*FAM161A*	0.68 [0.56,0.81]	1.58×10^{-5}	0.7 [0.58,0.86], p=4.66×10^{-4}	0.66 [0.54,0.81], p=4.74×10^{-5}	0.68 [0.56,0.82], p=6.60×10^{-5}	0.69 [0.57,0.85], p=3.35×10^{-4}	0.72 [0.58,0.89], p=0.003	0.7 [0.58,0.84], p=1.68×10^{-4}	0.7 [0.57,0.85], p=2.85×10^{-4}
22:21002277	rs369250	*THAP7*	1.44 [1.2,1.72]	1.50×10^{-5}	1.43 [1.18,1.73], p=2.47×10^{-4}	1.42 [1.17,1.72], p=4.33×10^{-4}	1.41 [1.17,1.71], p=2.85×10^{-4}	1.41 [1.16,1.71], p=4.50×10^{-4}	1.34 [1.09,1.66], p=0.007	1.44 [1.2,1.73], p=7.68×10^{-5}	1.42 [1.17,1.71], p=3.14×10^{-4}
7:101161149	rs1048365	*AP1S1*	1.73 [1.34,2.22]	2.07×10^{-5}	1.75 [1.33,2.31], p=7.22×10^{-5}	1.96 [1.49,2.59], p=1.88×10^{-6}	1.86 [1.42,2.44], p=6.90×10^{-6}	1.91 [1.45,2.52], p=3.95×10^{-6}	1.72 [1.27,2.34], p=4.57×10^{-4}	1.82 [1.4,2.36], p=7.93×10^{-6}	1.76 [1.34,2.31], p=4.83×10^{-5}
7:105107452	rs117986340	*KMT2E*	0.58 [0.45,0.74]	2.71×10^{-5}	0.61 [0.45,0.82], p=0.001	0.56 [0.42,0.76], p=1.70×10^{-4}	0.58 [0.43,0.77], p=2.17×10^{-4}	0.58 [0.43,0.79], p=5.07×10^{-4}	0.55 [0.4,0.76], p=3.36×10^{-4}	0.56 [0.42,0.75], p=8.67×10^{-5}	0.58 [0.43,0.77], p=2.75×10^{-4}
9:109176646	rs10979729	*EPB41L4B*	2.09 [1.53,2.87]	6.76×10^{-6}	2.15 [1.53,3.01], p=1.02×10^{-4}	2.06 [1.47,2.91], p=3.41×10^{-4}	2.07 [1.49,2.88], p=1.65×10^{-5}	2.08 [1.48,2.93], p=2.412e-05	2.22 [1.51,3.24], p=4.18×10^{-5}	2.11 [1.53,2.92], p=5.57×10^{-6}	2.12 [1.52,2.96], p=1.08×10^{-5}
**Gene aggregate tests**											
17:75522195*	rs200434678	*TSEN54*	11.57 [0.21, 633]	0.042	3.79 [0.08,178], p=0.497	27.4 [0.74,1011], p=0.07216	26.69 [0.79,901], p=0.06735	5.34 [0.12,235], p=0.3856	22.4 [0.34,1496], p=0.147	21.3 [1.1,412], p=0.043	51.94 [2.13,1267], p=0.015
17:75523335	rs200228117	*TSEN54*	4.88 [0.08, 304]	0.025	20.02 [2.46,163], p=0.005	8.07 [0.97,67], p=0.053	8.51 [1.04,69], p=0.046	6.7 [0.86,52], p=0.070	5.75 [0.46,72], p=0.175	10.43 [1.33,82], p=0.025	8.51 [1.18,62], p=0.034
4:86849099	rs17694522	*SLC10A6*	0.52 [0.37,0.73]	1.78×10^{-4}	0.53 [0.37,0.75], p=4.72×10^{-4}	0.57 [0.39,0.82], p=0.003	0.52 [0.36,0.74], p=3.01×10^{-4}	0.54 [0.38,0.79], p=0.001	0.55 [0.37,0.82], p=0.003	0.56 [0.39,0.79], p=0.001	0.58 [0.4,0.84], p=0.003
6:31572980	rs2229092	*LTA*	0.39 [0.24, 0.63]	1.48×10^{-4}	0.38 [0.23,0.64], p=2.37×10^{-4}	0.42 [0.25,0.7], p=0.0008824	0.39 [0.24,0.65], p=2.76×10^{-4}	0.44 [0.26,0.74], p=0.002	0.42 [0.24,0.74], p=0.002	0.42 [0.25,0.7], p=7.97×10^{-4}	0.37 [0.22,0.63], p=1.90×10^{-4}
7:101174620	rs34985488	*NAT16*	1.89 [1.38, 2.57]	5.85×10^{-5}	1.82 [1.29,2.56], p=6.51×10^{-4}	2.08 [1.48,2.93], p=2.276e-05	2.1 [1.51,2.93], p=1.15×10^{-5}	2.11 [1.5,2.95], p=1.457e-05	1.9 [1.3,2.76], p=8.29×10^{-4}	2.06 [1.49,2.83], p=1.11×10^{-4}	1.95 [1.39,2.71], p=8.95×10^{-5}
**Sliding windows and regulatory aggregate analysis**											
18:2351193*	rs16943099	*METTL4*	0.12 [0.04, 0.38]	2.09×10^{-4}	0.21 [0.09,0.5], p=4.35×10^{-4}	0.22 [0.09,0.53], p=8.25×10^{-4}	0.21 [0.08,0.51], p=656×10^{-4}	0.19 [0.07,0.46], p=3.04×10^{-4}	0.21 [0.08,0.55], p=0.001	0.17 [0.07,0.41], p=109×10^{-4}	0.19 [0.08,0.48], p=4.15×10^{-4}
2:40755782*	rs183413211	*LINC01794*	0.09 [0.02,0.48]	0.005	0.17 [0.03,1.04], p=0.055	0.07 [0.01,0.5], p=0.008	0.11 [0.02,0.81], p=0.030	0.06 [0.01,0.49], p=0.008185	0.08 [0.01,0.75], p=0.027	0.09 [0.01,0.57], p=0.011	0.06 [0.01,0.44], p=0.006
4:75124427*	rs114761270	-	5.13 [1.14, 202]	0.040	16.78 [2.77,102], p=0.002	17.27 [2.04,146], p=0.009	6.36 [0.83,49], p=0.075	7.27 [1.48,36], p=0.015	7.46 [1.4,40], p=0.018	7.55 [1.54,37], p=0.013	7.91 [1.55,40], p=0.013
9:87792152*	rs34407883	*CTSL*	0.33 [0.16, 0.69]	0.003	0.35 [0.17,0.74], p=0.005	0.36 [0.17,0.75], p=0.007	0.35 [0.17,0.73], p=0.0051	0.31 [0.14,0.67], p=0.003	0.25 [0.11,0.6], p=0.002	0.33 [0.16,0.67], p=0.002	0.31 [0.15,0.65], p=0.002
9:87792755*	rs12349827	*CTSL*	0.13 [0.02, 0.82]	0.030	0.08 [0.01,0.6], p=0.002	0.06 [0.01,0.32], p=8.67×10^{-4}	0.06 [0.01,0.29], p=5.82×10^{-4}	0.18 [0.03,1.21], p=0.077	0.14 [0.02,1.13], p=0.065	0.14 [0.03,0.68], p=0.015	0.22 [0.03,1.55], p=0.129
9:87793063*	rs12349926	*CTSL*	0.14 [0.05, 0.43]	6.08×10^{-4}	0.14 [0.03,0.6], p=0.008	0.11 [0.04,0.29], p=9.96×10^{-6}	0.09 [0.04,0.25], p=1.84×10^{-6}	0.15 [0.05,0.46], p=8.45×10^{-4}	0.08 [0.02,0.28], p=6.20×10^{-5}	0.18 [0.07,0.47], p=5.22×10^{-4}	0.17 [0.06,0.51], p=0.002

DKD OR/P-value: The original OR and p-values for association with DKD (adjusted for sex, calendar year of diabetes onset, and two first genetic principal components) are given for the reference. Further columns indicate OR [95% CI], p-value after adjusting for additional clinical covariates.

* Variant was only available in WGS data

[+]Included covariates: HbA_{1c}, diastolic blood pressure, systolic blood pressure, total cholesterol, triglycerides, BMI and smoking status

All analyses are additionally adjusted to for sex, age of type 1 diabetes onset and two most important genetic principal components

ESM Table 13 – The 17 lead variants from single variant, gene aggregate, sliding-window and regulome analysis tested for phenotype*sex interaction and more severe ESKD phenotype

Position	rsid	Gene*	DKD OR (*n*=1064)	*p* DKD (*n*=1064)	Sex × DKD interaction (*n*=1064)	OR ESKD (*n*=939)	*p* ESKD (*n*=939)
**SINGLE VARIANT ANALYSIS**							
2:61825245	rs3736598	*FAM161A*	0.67 [0.56,0.81]	1.30×10^{-5}	NS	0.75 [0.66, 0.87]	5.60×10^{-5}
2:61826155	rs6748320	*FAM161A*	0.68 [0.56,0.81]	1.58×10^{-5}	NS	0.76 [0.66, 0.87]	6.64×10^{-5}
22:21002277	rs369250	*THAP7*	1.44 [1.2,1.72]	1.50×10^{-5}	NS	1.31 [1.15, 1.5]	6.46×10^{-5}
7:101161149	rs1048365	*AP1S1*	1.73 [1.34,2.22]	2.07×10^{-5}	NS	1.54 [1.27, 1.88]	1.31×10^{-5}
7:105107452	rs117986340	*KMT2E*	0.58 [0.45,0.74]	2.71×10^{-5}	NS	0.64 [0.52, 0.78]	1.66×10^{-5}
9:109176646	rs10979729	*EPB41L4B*	2.09 [1.53,2.87]	6.76×10^{-6}	NS	1.67 [1.31, 2.14]	3.82×10^{-5}
**GENE AGGREGATE ANALYSIS**							
17:75522195*	rs200434678	*TSEN54*	11.57 [0.21, 633]	0.042	NS	4.97 [0.62, 39]	0.130
17:75523335	rs200228117	*TSEN54*	4.88 [0.08, 304]	0.025	NS	5.84 [0.75, 45]	0,095
4:86849099	rs17694522	*SLC10A6*	0.52 [0.37,0.73]	1.78×10^{-4}	NS	0.66 [0.51,0.84]	0,001
6:31572980	rs2229092	*LTA*	0.39 [0.24, 0.63]	1.48×10^{-4}	NS	0.51 [0.35, 0.73]	2.83×10^{-4}
7:101174620	rs34985488	*NAT16*	1.89 [1.38, 2.57]	5.85×10^{-5}	NS	1.64 [1.29,2.08]	5.07×10^{-5}
**SLIDING-WINDOW, PROMOTOR AND ENHANCER-WIDE ANALYSIS ON WGS DATA**							
18:2351193*	rs16943099	*METTL4*	0.12 [0.04, 0.38]	2.09×10^{-4}	0.01	0.33 [0.18, 0.61]	3.58×10^{-4}
2:40755782*	rs183413211	*LINC01794*	0.09 [0.02,0.48]	0.005	NS	0.24 [0.07, 0.8]	0.020
4:75124427*	rs114761270	-	5.13 [1.14, 202]	0.040	NS	5.13 [1.54, 17.13]	0.008
9:87792152*	rs34407883	*CTSL*	0.33 [0.16, 0.69]	0.003	NS	0.51 [0.31, 0.83]	0.006
9:87792755*	rs12349827	*CTSL*	0.13 [0.02, 0.82]	0.030	NS	0.35 [0.12, 0.99]	0.048
9:87793063*	rs12349926	*CTSL*	0.14 [0.05, 0.43]	6.08×10^{-4}	NS	0.35 [0.19, 0.65]	0.001

* Variant was only available in WGS data

DKD is the original phenotype of the study, in which cases have either either severe albuminruia or ESKD

ESM Table 14 - FinnDiane physicians and nurses participating in the collection of the FinnDiane study subjects

FinnDiane Study Centers	Physicians and nurses
Anjalankoski Health Centre	S. Koivula, T. Uggeldahl
Central Finland Central Hospital, Jyväskylä	T. Forslund, A. Halonen, A. Koistinen, P. Koskiaho, M. Laukkanen, J. Saltevo, M. Tiihonen
Central Hospital of Åland Islands, Mariehamn	M. Forsen, H. Granlund, A-C. Jonsson, B. Nyroos
Central Hospital of Kanta-Häme, Hämeenlinna	P. Kinnunen, A. Orvola, T. Salonen, A. Vähänen
Central Hospital of Länsi-Pohja, Kemi	H. Laukkanen, P. Nyländen, A. Sademies
Central Ostrabothnian Hospital District, Kokkola	S. Anderson, B. Asplund, U. Byskata, P. Liedes, M. Kuusela, T. Virkkala
City of Espoo Health Centre	
Espoonlahti	A. Nikkola, E. Ritola
Tapiola	M. Niska, H. Saarinen
Samaria	E. Oukko-Ruponen, T. Virtanen
Viherlaakso	A. Lyytinen
City of Helsinki Health Centre	
Puistola	H. Kari, T. Simonen
Suutarila	A. Kaprio, J. Kärkkäinen, B. Rantaeskola
Töölö	P. Kääriäinen, J. Haaga, A-L. Pietiläinen
City of Hyvinkää Health Centre	S. Klemetti, T. Nyandoto, E. Rontu, S. Satuli-Autere
City of Vantaa Health Centre	
Korso	R. Toivonen, H. Virtanen
Länsimäki	R. Ahonen, M. Ivaska-Suomela, A. Jauhiainen
Martinlaakso	M. Laine, T. Pellonpää, R. Puranen
Myyrmäki	A. Airas, J. Laakso, K. Rautavaara
Rekola	M. Erola, E. Jatkola
Tikkurila	R. Lönnblad, A. Malm, J. Mäkelä, E. Rautamo
Heinola Health Centre	P. Hentunen, J. Lagerstam
Helsinki University Central Hospital, Department of Medicine, Division of Nephrology	A. Ahola, J. Fagerudd, M. Feodoroff, D. Gordin, O. Heikkilä, K Hietala, L. Kyllönen, J. Kytö, S. Lindh, K. Pettersson-Fernholm, M. Rosengård-Bärlund, M. Rönnback, A. Sandelin, A-R Salonen, L. Salovaara, L. Thorn, J. Tuomikangas, T. Vesisenaho, J. Wadén
Herttoniemi Hospital, Helsinki	V. Sipilä
Hospital of Lounais-Häme, Forssa	T. Kalliomäki, J. Koskelainen, R. Nikkanen, N. Savolainen, H. Sulonen, E. Valtonen
Iisalmi Hospital	E. Toivanen
Jokilaakso Hospital, Jämsä	A. Parta, I. Pirttiniemi

FinnDiane Study Centers	Physicians and nurses
Jorvi Hospital, Helsinki University Central Hospital	S. Aranko, S. Ervasti, R. Kauppinen-Mäkelin, A. Kuusisto, T. Leppälä, K. Nikkilä, L. Pekkonen
Jyväskylä Health Centre, Kyllö	K. Nuorva, M. Tiihonen
Kainuu Central Hospital, Kajaani	S. Jokelainen, P. Kemppainen, A-M. Mankinen, M. Sankari
Kerava Health Centre	H. Stuckey, P. Suominen
Kirkkonummi Health Centre	A. Lappalainen, M. Liimatainen, J. Santaholma
Kivelä Hospital, Helsinki	A. Aimolahti, E. Huovinen
Koskela Hospital, Helsinki	V. Ilkka, M. Lehtimäki
Kotka Heath Centre	E. Pälikkö-Kontinen, A. Vanhanen
Kouvola Health Centre	E. Koskinen, T. Siitonen
Kuopio University Hospital	E. Huttunen, R. Ikäheimo, P. Karhapää, P. Kekäläinen, M. Laakso, T. Lakka, E. Lampainen, L. Moilanen, L. Niskanen, U. Tuovinen, I. Vauhkonen, E. Voutilainen
Kuusamo Health Centre	T. Kääriäinen, E. Isopoussu
Kuusankoski Hospital	E. Kilkki, I. Koskinen, L. Riihelä
Laakso Hospital, Helsinki	T. Meriläinen, P. Poukka, R. Savolainen, N. Uhlenius
Lahti City Hospital	A. Mäkelä, M. Tanner
Lapland Central Hospital, Rovaniemi	L. Hyvärinen, S. Severinkangas, T. Tulokas
Lappeenranta Health Centre	P. Linkola, I. Pulli
Lohja Hospital	T. Granlund, M. Saari, T. Salonen
Loimaa Health Centre	A. Mäkelä, P. Eloranta
Länsi-Uusimaa Hospital, Tammisaari	I-M. Jousmaa, J. Rinne
Malmi Hospital, Helsinki	H. Lanki, S. Moilanen, M. Tilly-Kiesi
Mikkeli Central Hospital	A. Gynther, R. Manninen, P. Nironen, M. Salminen, T. Vänttinen
Mänttä Regional Hospital	I. Pirttiniemi, A-M. Hänninen
North Karelian Hospital, Joensuu	U-M. Henttula, P. Kekäläinen, M. Pietarinen, A. Rissanen, M. Voutilainen
Nurmijärvi Health Centre	A. Burgos, K. Urtamo
Oulankangas Hospital, Oulainen	E. Jokelainen, P-L. Jylkkä, E. Kaarlela, J. Vuolaspuro
Oulu Health Centre	L. Hiltunen, R. Häkkinen, S. Keinänen-Kiukaanniemi
Oulu University Hospital	R. Ikäheimo
Päijät-Häme Central Hospital	H. Haapamäki, A. Helanterä, S. Hämäläinen, V. Ilvesmäki, H. Miettinen
Palokka Health Centre	P. Sopanen, L. Welling
Pieksämäki Hospital	V. Javtsenko, M. Tamminen
Pietarsaari Hospital	M-L. Holmbäck, B. Isomaa, L. Sarelin
Pori City Hospital	P. Ahonen, P. Merensalo, K. Sävelä
Porvoo Hospital	M. Kallio, B. Rask, S. Rämö
Raahe Hospital	A. Holma, M. Honkala, A. Tuomivaara, R. Vainionpää
Rauma Hospital	K. Laine, K. Saarinen, T. Salminen
Riihimäki Hospital	P. Aalto, E. Immonen, L. Juurinen

FinnDiane Study Centers	Physicians and nurses
Salo Hospital	A. Alanko, J. Lapinleimu, P. Rautio, M. Virtanen
Satakunta Central Hospital, Pori	M. Asola, M. Juhola, P. Kunelius, M-L. Lahdenmäki, P. Pääkkönen, M. Rautavirta
Savonlinna Central Hospital	E. Korpi-Hyövälti, T. Latvala, E. Leijala
South Karelia Central Hospital, Lappeenranta	T. Ensala, E. Hussi, R. Härkönen, U. Nyholm, J. Toivanen
Tampere Health Centre	A. Vaden, P. Alarotu, E. Kujansuu, H. Kirkkopelto-Jokinen, M. Helin, S. Gummerus, L. Calonius, T. Niskanen, T. Kaitala, T. Vatanen
Tampere University Hospital	I. Ala-Houhala, T. Kuningas, P. Lampinen, M. Määttä, H. Oksala, T. Oksanen, K. Salonen, H. Tauriainen, S. Tulokas
Tiirismaa Health Centre, Hollola	T. Kivelä, L, Petlin, L. Savolainen
Turku Health Centre	I. Hämäläinen, H. Virtamo, M. Vähätalo
Turku University Central Hospital	K. Breitholz, R. Eskola, K. Metsärinne, U. Pietilä, P. Saarinen, R. Tuominen, S. Äyräpää
Vaajakoski Health Centre	K. Mäkinen, P. Sopanen
Valkeakoski Regional Hospital	S. Ojanen, E. Valtonen, H. Ylönen, M. Rautiainen, T. Immonen
Vammala Regional Hospital	I. Isomäki, R. Kroneld, M. Tapiolinna-Mäkelä
Vaasa Central Hospital	S. Bergkulla, U. Hautamäki, V-A. Myllyniemi, I. Rusk

REFERENCES

[1] Wilson PC, Wu H, Kirita Y, et al. (2019) The single-cell transcriptomic landscape of early human diabetic nephropathy. Proceedings of the National Academy of Sciences 116: 19619-19625
[2] Woroniecka KI, Park ASD, Mohtat D, Thomas DB, Pullman JM, Susztak K (2011) Transcriptome analysis of human diabetic kidney disease. Diabetes 60: 2354-2369
[3] Li Q, Wang K (2017) InterVar: clinical interpretation of genetic variants by the 2015 ACMG-AMP guidelines. The American Journal of Human Genetics 100: 267-280
[4] Schmidt RJ, Steeves M, Bayrak-Toydemir P, et al. (2024) Recommendations for risk allele evidence curation, classification, and reporting from the ClinGen Low Penetrance/Risk Allele Working Group. Genetics in Medicine 26: 101036
[5] Liu H, Doke T, Guo D, et al. (2022) Epigenomic and transcriptomic analyses define core cell types, genes and targetable mechanisms for kidney disease. Nature Genetics 54: 950-962
[6] Erlich H, Valdes AM, Noble J, et al. (2008) HLA DR-DQ haplotypes and genotypes and type 1 diabetes risk: analysis of the type 1 diabetes genetics consortium families
